# The Role of Water in the Adsorption of Nitro-Organic Pollutants on Activated Carbon

**DOI:** 10.1021/acs.jpca.3c03877

**Published:** 2023-09-25

**Authors:** Celia Adjal, Vicente Timón, Nabila Guechtouli, Rahma Boussassi, Dalila Hammoutène, María Luisa Senent

**Affiliations:** † Laboratory of Thermodynamics and Molecular Modeling, Faculty of Chemistry, USTHB, BP32, El Alia, Bab Ezzouar,Algiers 16111, Algeria; ‡ Instituto de Estructura de la Materia, CSIC, Serrano 121, Madrid 28006, Spain; § Faculty of Sciences, Department of Chemistry, Mouloud Mammeri University of Tizi Ouzou, UMMTO, Tizi Ouzou 15000, Algeria

## Abstract

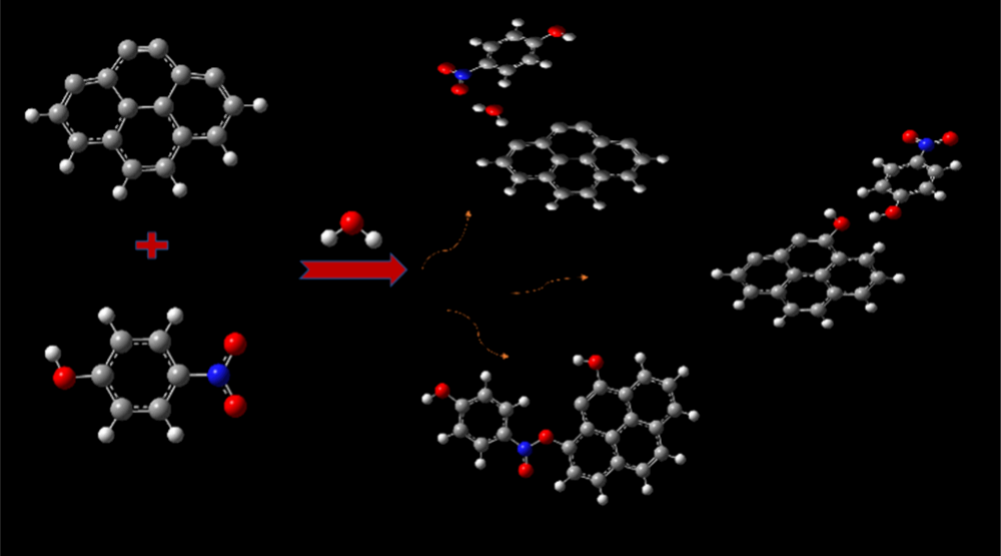

The density functional theory (DFT) is applied to theoretically study the capture and storage of three different nitro polycyclic aromatic hydrocarbons, 4-nitrophenol, 2-nitrophenol, and 9-nitroanthracene by activated carbon, with and without the presence of water. These species are pollutants derived from vehicle and industry emissions. The modeling of adsorption is carried out at the molecular level using a high-level density functional theory with the B3LYP-GD(BJ)/6-31+G(d,p) level of theory. The adsorption energies of polluting gases considered isolated and in a humid environment are compared to better understand the role of water. The calculations reveal different possible pathways involving the formation of chemical bonds between adsorbent and adsorbate on the formation of intermolecular van der Waals interactions. The negative adsorption energy on AC for the three species is obtained when they are treated individually and in mixture with H_2_O. The basis-set superposition error, estimated using the counterpoise correction, varies the adsorption energies by 2–13%. Dispersion effects were also taken into account. The adsorption energy ranges from −10 to −414 kJ/mol suggesting a diversity of pathways. The resulting analysis suggests three preferred pathways for capture. The main pathway is physical interaction due to π–π stacking. Other means are capture due to the formation of hydrogen bonds resulting from water adsorbed on the surface and the simultaneous adsorption of pollutant and water where water can act as a link that promotes adsorption. The thermodynamic properties give a clue to the most eco-friendly approaches for molecular adsorption.

## 1 Introduction

Vehicle emissions have increased very rapidly around the world, ignoring the health consequences and effects on wildlife and plants at great risk of extinction. Otherwise, many water sources are contaminated, and wastewater has increased dramatically in the past few years. Polycyclic aromatic compounds (PACs) are the main group of gas pollutants from car exhaust that generate polycyclic aromatic hydrocarbons (PAHs) and oxygenated (OPAHs) and nitro polycyclic aromatic hydrocarbons (NPAHs) derivatives. Many of these chemicals are classified as volatile organic compounds (VOCs).

NPAHs or nitro-organic compounds are derived from polycyclic aromatic, and the incomplete combustion of fossil fuels is significant sources of these chemicals.^1−4^

A variety of NPAHs, the nitrophenols, are worthy hazardous pollutants found in our daily life.^5,6^ They can be synthesized by following photochemical reactions between benzene and nitrogen monoxide in highly polluted air. Even at low concentrations and due to their structure and physicochemical properties, they are able to contribute to the diver’s effect on aquatic fauna and flora because they contribute significantly to water contamination. This is why the United States Environmental Protection Agency has classified 4-nitrophenol (4NP) and 2-nitrophenol (2NP) as “priority pollutants”,^7^ and the European Union (EU) has promulgated regulations for PAHs in water.^8^

So far, low concentrations of NPAHs have been found in the environment compared to PAHs, but recently, high concentrations have been observed in China, Russia, Korea, and Japan.^9^ For example, the NPAH 9-nitroanthracene (9NAnt) has been consistently detected at a concentration of 350 ng L^–1^ causing numerous pathological response and DNA damage.^10^

Different methods, such as membrane filtration, advanced oxidation, enzymatic treatment, chromatography, or adsorption on solid surfaces, are applied in searching for cleaning the atmosphere. The techniques based on chemical and physical adsorption are those considered in the present work.^11−13^ The adsorption mechanism occurs by adhesion of gases called a substrate or adsorbent on the surface of a solid called adsorbent.^14^ This method is considered by many scientists because it is relatively simple, and it is low-cost.^15^ Moreover, it is effective for selective separation, even in the presence of very small amounts of substrate.

Many adsorbents are used nowadays, such us MOFs^16^ or ZIFs,^17,18^ bentonite clay^19^ and silica,^20^ different types of activated carbon (AC), and many other filters. They are chosen because of their physicochemical behavior and efficiency for the capture and storage of pollutants.

Previous studies have revealed the suitability of AC for the capture of PAHs with many technical applications.^5,6^ Activated carbon is characterized by its highly developed porosity and large surface area. In addition, AC is a very popular low-cost material responsible for the adsorption capacity. Its chemical structure interacts with polar and nonpolar adsorbates.^21^

This is the reason why, following the previous experiments^17,25^ in this paper, we treat at the molecular level the ability of AC for the capture of 2NP, 4NP, and 9NAnt using the density functional theory. AC is represented by a complex aromatic structure of 16 carbon and 6 hydrogen atoms. Previous studies on the adsorption of greenhouse gases by ZIFs performed using molecular and solid-state models show the ability of molecular calculations to predict adsorption properties.^17,25^

In addition, the effect of water is described by adding water molecules instead of using an electrostatic model that is not powerful enough to determine the effects of the geometry distortion due to water. Despite the relevant toxicity of 2-nitrophenols, few previous theoretical and experimental studies have addressed these pollutants, perhaps due to their low abundance. Few previous papers have been devoted to capture and storage. The results of the present paper represent predictions that may be useful for future laboratory studies.

## 2 Computational Details

In this paper, adsorption of 4NP, 2NP, and 9Nant nitro-organic compounds on activated carbon (AC) was simulated using electronic structure methodology applied to molecules. All calculations were performed using Gaussian16 suite.^22^ Thermodynamic properties were computed under ambient temperature and pressure conditions. The density functional theory (DFT) was applied to study the interactions between the nitro-organic pollutants and adsorption surface. The optimization of the stable ground electronic state geometries, the calculations of the corresponding energies, harmonic frequencies, and thermodynamic properties were carried out using the B3LYP hybrid functional.^25^ This includes Becke’s parameter exchange functional (B3)^62,63^ and the Lee, Yang, Parr (LYP) gradient-corrected correlation functional.^25−61^ At first, the functional was applied in connection with the 6-31G basis set to obtain a preliminary description of the reaction pathways. The final results were obtained using the 6-31+G(d,p) basis set, which contains diffuse orbitals capable of describing long-range interactions.^23,24^ To empirically consider a van der Waals (vdW) dispersion interaction involving adsorbate, pollutants, and water, we apply the B3LYP-GD3(BJ) functional. Grimme’s dispersion correction is the most popular way to deal with adsorption cases of weakly bounded systems, where long-range effects must be considered.^25−27^ In addition, the BSSE error was estimated using the counterpoise keyword implemented in Gaussian software as a correction to refine the computed molecular parameter. This yields more reliable results by mitigating the error introduced by using separate basis sets for each molecule in the computation.^28−32^

Modeling at the molecular level, AC (the bulk) is represented by a complex aromatic structure of 16 carbon and 6 hydrogen atoms. Optimization was done without symmetry constraint.

The adsorption energy of a gas molecule *E*_ads_ (G) is calculated with the following equation:^33^
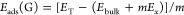
1where *E*_bulk_, *E*_*x*_, and *m* represent the energy of the AC bulk considered as isolated, the energy of one molecule of the substrate, and the number of adsorbed molecules. *E*_T_ refers to the total energy of the AC bulk with a molecule of gas adsorbed on the surface.

Our main objective is to compare the behavior in the presence or absence of water. Then, eq 1 is transformed to include *s*H_2_O molecules. Then,^33,34^
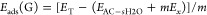
2

In this equation, *E*_T_ represents the total energy of AC, water, and pollutant, while *E*_AC+*s*H2O_ refers to a complex structure formed by water and AC; *E*_*x*_ deals with the energy of the pollutant. The adsorption energy recovered from eq 2 refers to the capture of pollutants by the AC-water entity.

An original subroutine allows us to determine the adsorption and interaction energies from the resulting DFT energies computed with Gaussian16. The code, which is provided in the Supporting Information, was written using the PYTHON programming language. Following a general criterion,^35^ we interpret as chemisorption when the adsorption energy is greater than −50 kJ/mol and physisorption when the adsorption energy is less than −30 kJ/mol. Physisorption occurs due to electrostatic interactions, weak van der Waals interactions, hydrophobic and hydrophilic interactions, π–π stacking interactions,^36,37^ and hydrogen bounds.^38−40^ We classified hydrogen bonds according to bond lengths. When the bond distances range between 1.2 and 1.5 Å, 1.5 and 2.2 Å, and 2.2 and 3.0 Å, we consider that the intermolecular interaction is, respectively, very strong, strong, and moderately strong.

## 3 Results and Discussion

The first step of this work was the description of the molecular structures representing the solid activated carbon adsorbent as well as those of the adsorbates. In previous theoretical works, the suitability of different molecular structures such as graphite crystal structures, benzene ring cluster models, has been verified.^35−41^ For this work, an armchair model of pyrene with four benzene rings was selected and modified to obtain a final C_16_H_6_ structure.^42−44^

In a second step, complex structures were derived from the approach of one molecule of 2NP, 4NP, 9NAnt, and water to the bulk. The third step is the study of the coadsorption of water and pollutants to determine the role played by humidity. In a fourth step, a second pollutant molecule is added to the previously computed complexes. The last step concerns a thermodynamic study.

### 3.1 Isolated Structures of the Activated Carbon and the Pollutants

**Figure 1:**
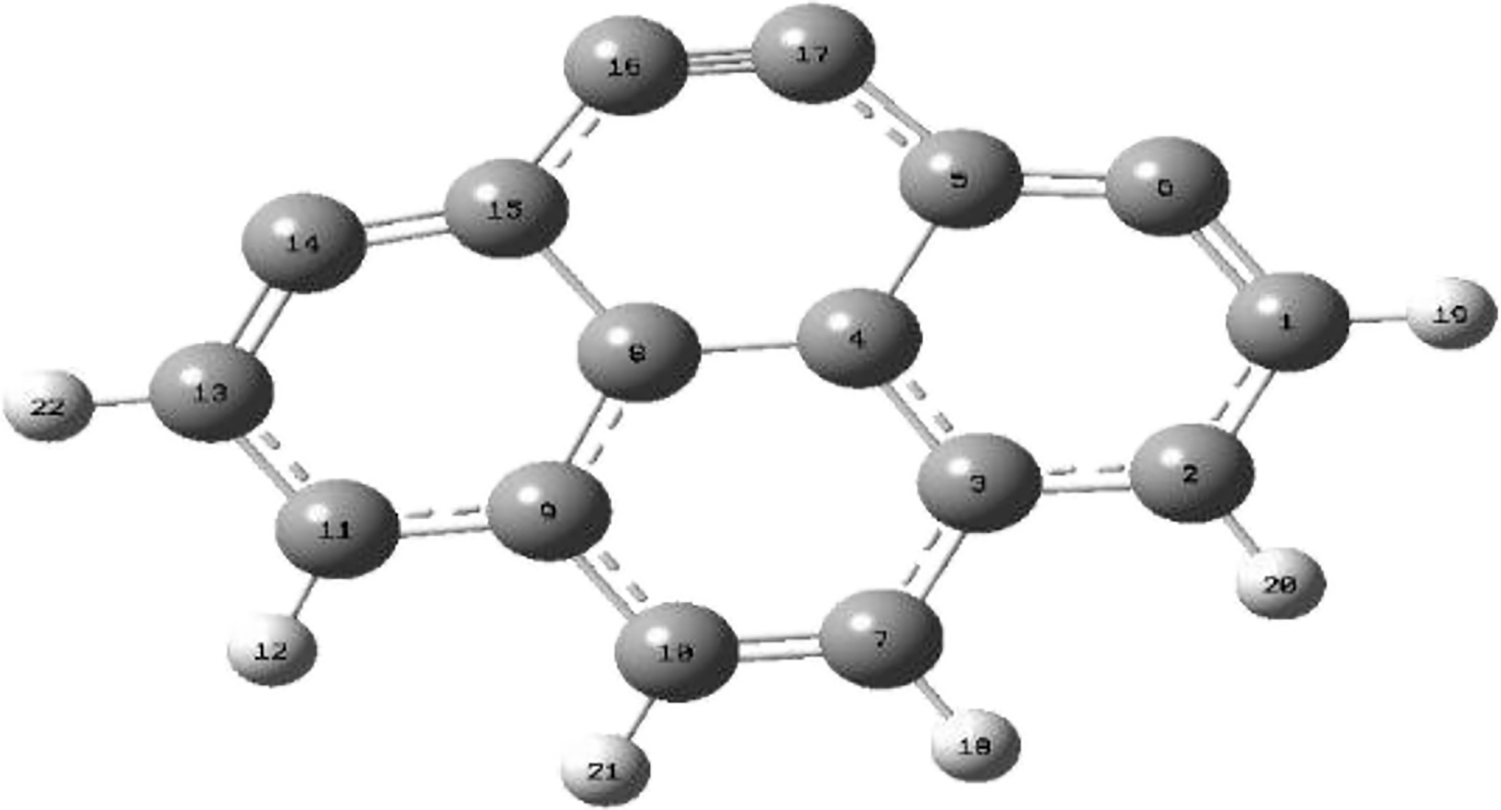
Optimized molecular structure employed for the simulation of activated carbon.

The AC model used in our study was extracted from the armchair mode of pyrene collected in the Gaussian database^5^ by unsaturating the upper side carbon atoms to get the C_16_H_6_ bulk. The resulting structure of geometry optimization is shown in Figure 1. Table 1 contains the optimized structural parameters computed using DFT/6-31+G(d,p), which are provided in the Supporting Information. The pollutants have also been optimized at the same level of theory, and the structural parameters are also provided in the Supporting Information.

**Table 1:** B3LYP/6-31+G(d,p) Structural Parameters of AC, Considered to be Isolated and in the Presence of One-Adsorbed Molecule of Water, 4NP, 2NP, or 9NAnt

			AC-4NP		AC-2NP		
bond distances(Å)	AC	AC-water	OH	NO_2_	OH	NO_2_	AC-9NAnt
C_17_–C_16_	1.241	1.334	1.247	1.391	1.243	1.242	1.336
C_16_–C_15_	1.418	1.444	1.436	1.424	1.423	1.417	1.417
C_5_–C_17_	1.418	1.405	1.410	1.398	1.415	1.418	1.431
C_14_–C_15_	1.365	1.407	1.359	1.473	1.395	1.366	1.368
C_5_–C_6_	1.365	1.369	1.361	1.417	1.381	1.369	1.389
C_8_–C_15_	1.468	1.422	1.463	1.422	1.446	1.468	1.499
C_5_–C_4_	1.468	1.496	1.472	1.471	1.477	1.467	1.395
C_6_–C_1_	1.369	1.369	1.368	1.383	1.389	1.373	1.397
C_13_–C_14_	1.369	1.369	1.369	1.459	1.392	1.369	1.368
C_4_–C_3_	1.432	1.436	1.432	1.429	1.428	1.432	1.429
C_8_–C_9_	1.432	1.436	1.433	1.430	1.432	1.432	1.430
C_13_–C_11_	1.401	1.392	1.401	1.362	1.392	1.401	1.399
C_1_–C_2_	1.401	1.397	1.400	1.313	1.397	1.339	1.403
C_3_–C_7_	1.438	1.441	1.438	1.421	1.437	1.436	1.439
C_9_–C_10_	1.438	1.432	1.437	1.401	1.436	1.436	1.445
C_15_C_16_C_17_	128.8	118.1	123.8	121.4	121.2	128.5	127.5
C_5_C_16_C_17_	128.8	131.6	133.4	122.9	135.4	129.2	117.4
C_17_C_5_C_6_	136.4	122.3	138.2	128.8	134.5	136.0	113.6
C_16_C_15_C_14_	136.4	119.9	134.7	122.3	127.8	136.4	137.4
C_14_C_13_C_22_	122.7	120.1	123.2	116.6	119.7	122.8	121.3
C_6_C_1_C_19_	122.7	121.4	122.8	120.8	120.7	122.1	121.4

For isolated AC, our preliminary results computed using B3LYP/6-31G (i.e., C_17_–C_16_ = 1.247 Å; C_16_–C_15_ = 1.422 Å) are in a good agreement with the previous ones of Supong et al.^5,42^ computed before adsorption at the B3LYP/6-31G level of theory (C_17_–C_16_ = 1.248 Å; C_16_–C_15_ = 1.425 Å). These authors simulated the adsorption effect on the geometry locating AC in a humid environment described by an electrostatic model (ε(water) = 80) implemented in Gaussian. Since our geometry is computed at the same level of theory considering that the AC is totally isolated, the agreement between our calculations and those of refs^5,42^ reveals the limitations of the electrostatic model in accurately describing molecular distortion caused by humidity. This is the reason why, in this paper, water is represented by a set of *s*H_2_O molecules.

The optimized isolated surface is a slightly distorted C_2v_ structure featuring four benzene rings. Symmetry is evident in internal coordinates Table 1, e.g., C_5_–C_17_/C_15_-C_16_ bond pair (1.418 Å, 128.8°) and C_3_–C_7_/C_9_-C_10_ pair (length = 1.438 Å) with angles C_14_C_13_C_22_/C_6_C_1_C_19_ (122.6°). Dihedral angles such as C_15_C_16_C_17_C_5_ were found to be zero, which confirms the symmetry and flatness of the AC. Interactions with nitro-organic compounds or water molecules slightly distort the geometry, enlarging the C–C bonds on the surface.

The optimized parameters in Table 1 confirm that bulk distortion is more pronounced from the bottom to the top. The large variation corresponds to the chemisorbed structure, which occurs by breaking or forming chemical bonds, generated on the surface of the adsorbent.

### 3.2 2-Nitrophenol, 4-Nitrophenol, 9-Nitroanthracene, and Water Adsorption on Activated Carbon

**Figure 2:**
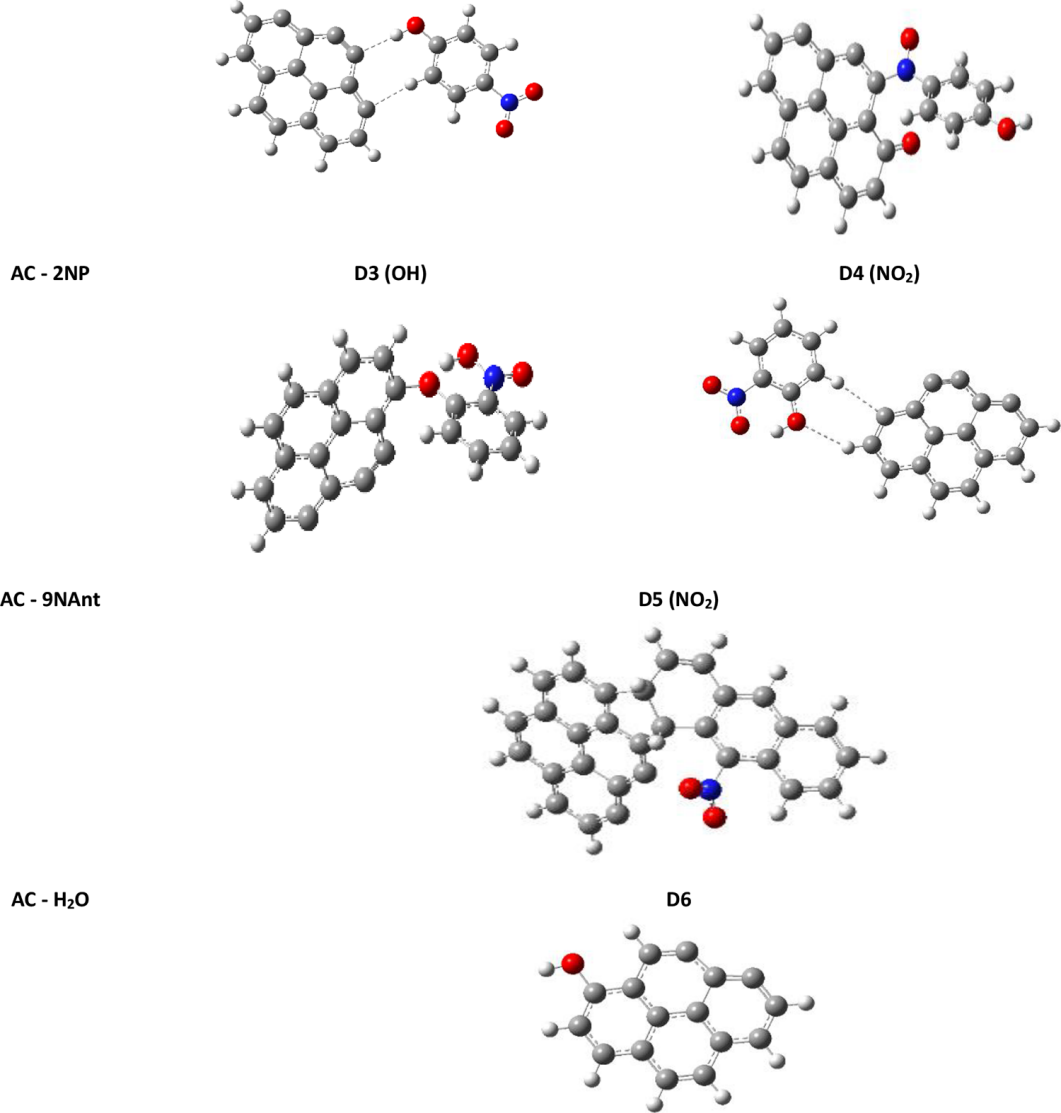
Optimized structures of pollutants and water linked to activated carbon to form aggregates of two molecules (dotted (---) refers to the physical interactions shown in Table 2).

To obtain the complex structures of the pollutants bound to the AC surface, the adsorbates were initially placed 3 Å from the bulk in many different orientations covering the whole space around AC. The pollutants were rotated to facilitate the link through the OH or the NO_2_ functional groups. We denote DX for dimers and TX for trimers (X is a configuration labeling). For example DX(OH) and DX(NO_2_) denotes two-body aggregates linked trough the OH and NO_2_ groups, respectively. This procedure allows one to carry out a systematic and complete search. As expected, the upper AC side, containing unsaturated carbon atoms, represents the most active region. Figure 2 illustrates the final optimized structures after a total unconstrained energy optimization.

Both 4NP and 2NP structures exhibit opposite behaviors, Figure 2. While 4NP is more reactive at the nitro-side, 2NP tends to be bulk bound on the hydroxyl side. Both lead to the creation of new bonds with chemisorption energies of −375 kJ/mol (4NP) and −180 kJ/mol (2NP), respectively, as shown in Table 2. We note that the adsorption energy of 4NP is more important than in 2NP, due to the number of bonds involved in the adsorption process. Indeed, for the D2 dimer, we report the creation of two new covalent bonds, one between an AC carbon and the oxygen atom of the NO_2_ group, and the other bond with an AC carbon and the nitrogen atom of 4NP. The NO_2_ group exhibits the highest chemical reactivity, as the nitrogen atom forms a strong covalent bond of 1.416 Å with the activated carbon. On the other hand, the NO_2_ oxygen atom links AC forming another bond of 1.247 Å. However, in D3, two covalent bonds are created. In the first one, the OH oxygen atom connects to one of the AC carbons. In the second one, an intramolecular bond is formed between the two functional groups of 2NP, which gives some stability to the compound.

**Table 2:** Adsorption Energies, Computed without (*E*_ads_) and within (*E*_adsGD3(BJ)_) Grimme’s Empirical Dispersion and Shorter Intermolecular Bond Distances between Pollutants and Activated Carbon^a^

*A*C+1 pollutant molecule or 1 water molecule		intermolecular bond distances (Å)		*E*_ads_ (kJ/mol)	*E*_adsGD3(BJ)_ (kJ/mol)	BSSE (kJ/mol)
**D1** (OH)	AC-4NP (OH)	(AC) C---HO (4NP)	2.114	–40	–39	2.62
		(AC) C---HC (4NP)	2.365			
**D2** (NO_2_)	AC-4NP (NO_2_)	(AC) C–NO_2_ (4NP)	1.416	–375	–396	10.50
		(AC) C–O_2_N (4NP)	1.247			
**D3** (OH)	AC-2NP (OH)	(AC) C–OH (2NP)	1.413	–180	–194	9.18
		(2NP) NO_2_–HO (2NP)	0.994			
**D4** (NO_2_)	AC-2NP (NO_2_)	(AC) C---HC (2NP)	2.596	–26	–383	1.31
		(AC) CH---OH (2NP)	2.602			
**D5** (NO_2_)	AC-9NAnt (NO_2_)	(AC) C–CH (9NAnt)	1.543	–384	–411	10.23
		(AC) C–CH (9NAnt)	1.528			
**D6**	AC-H_2_O	(AC) C–O (H_2_O)	1.369	–414	–409	9.45
		(AC) C–H (H_2_O)	1.087			

a Estimation of the superposition basis set error (BSSE) on the energy. Dotted (---) refers to the physical interactions, and direct line (−) refers to a covalent bound. The effect of the empirical dispersion on the distances is negligible.

For the last nitrophenol compounds D1 and D4, physisorption occurs without changes in the molecular structure, by weak bonds such as π-H-interactions, due to noncovalent attraction between the nitrophenolic rings and AC rings, and by other electrostatic interactions. A rotation of the phenolic group occurs to minimize the distance between the pollutant and bulk and maximize stacking. The π-H-interactions stabilizes and characterize D1 and D4. The corresponding distances of 2.365 Å (D1) and 2.596 Å (D4) are nearly twice the estimated van der Waals radius of carbon of 1.7 Å.

Furthermore, we note the presence of a C•••HO hydrogen bond of 2.114 Å in D1, stronger than its counterpart in the D4 system, which is 2.602 Å. This type of interaction is less intense in 2NP due to the hindrance between the two functional groups. Indeed, hydrogen bonding plays an important role in the adsorption of 4NP on AC, favoring physical adsorption.

In the D5 system, 9NAnt has a different behavior from the other studied pollutants; due to the high electron density, the three benzene rings perform an internal rotation breaking the plane of the activated carbon. This contributes to increase the carbon surface by the fusion of AC and 9NAnt rings, which easily makes the formation of two new C–C single bounds of 1.5 Å carries out a significant *E*_ads_ of −384 kJ/mol. Finally, the D6 complex arises from the adsorption of the water molecule on the upper surface of AC, which is done very easily due to the opposite density charge, leading to a higher adsorption energy with −414 kJ/mol. It may be concluded from these results that all of the studied pollutants can be attracted by AC.

Empirical dispersion applied for physisorption slightly increases the adsorption energies, while intramolecular bond distances decrease, causing pollutants and AC to come closer together. This distance variation, being more pronounced in the case of D4, transforms the links by physisorption into links by chemisorption and leads to the creation of a covalent bond between the 2NP and bulk. Indeed, D4 behaves like D3 because the two functional groups act as a single fragment of intense electron density, leading to a strong Coulomb interaction between the functional groups and AC.

In the case of chemisorption (D2, D3, D5, and D6), the adsorption energies are overestimated by 5% compared to the initial adsorption energy without BSSE correction. This is due to the absence of weak interactions. Indeed, the dispersion favors noncovalent interactions.

In these approaches, the effect on the energy of the BSSE correction is about 2–5%. The effect on geometry optimization is negligible and does not change the nature of adsorption.

### 3.3 Coadsorption of 2-Nitrophenol, 4-Nitrophenol, 9-Nitroanthracene, and Water on Activated Carbon

Particular attention is paid to the role of water and the effect of the atmosphere humidity on the efficiency of solid materials for gas capture. In many theoretical studies,^5,33^ this effect is taken into account using more or less sophisticated electrostatic models. However, as already explained, these models neglect effects such as coadsorption and do not well describe molecular distortions due to humidity. Then, in the present paper, water is represented by one H_2_O molecule, which is introduced simultaneously with pollutants.

**Figure 3:**
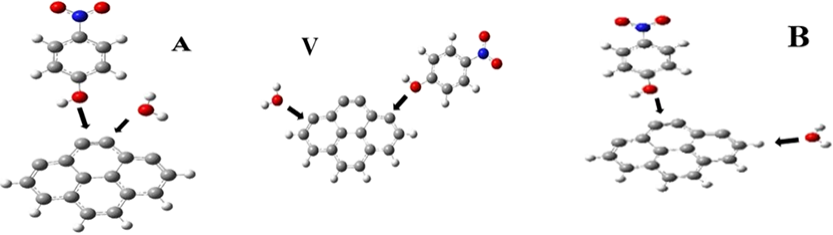
Three initial relative orientations of water and NAPH molecules considered for the simulation of the coadsorption processes.

As described above, to initiate a systematic search for optimized structures of the complexes, both nitrophenol and water molecules are placed 3 Å from the AC surface in selected orientations covering the entire physical space around the bulk. The two molecules are designed to approach each other from different relative orientations: (1) both attacks from the same side of the bulk (A-initial configuration), (2) the attack is from opposite sides of the bulk (V-initial configuration), and (3) the two molecules follow perpendicular pathways (B-initial configuration). The three approaches are represented in Figure 3. The selected initial configurations A-B discriminate the formation of complex structures by chemisorption or linked by physisorption. Configuration A favors the physisorption, while configurations V and B favor complete and partial chemisorption, respectively.

**Figure 4:**
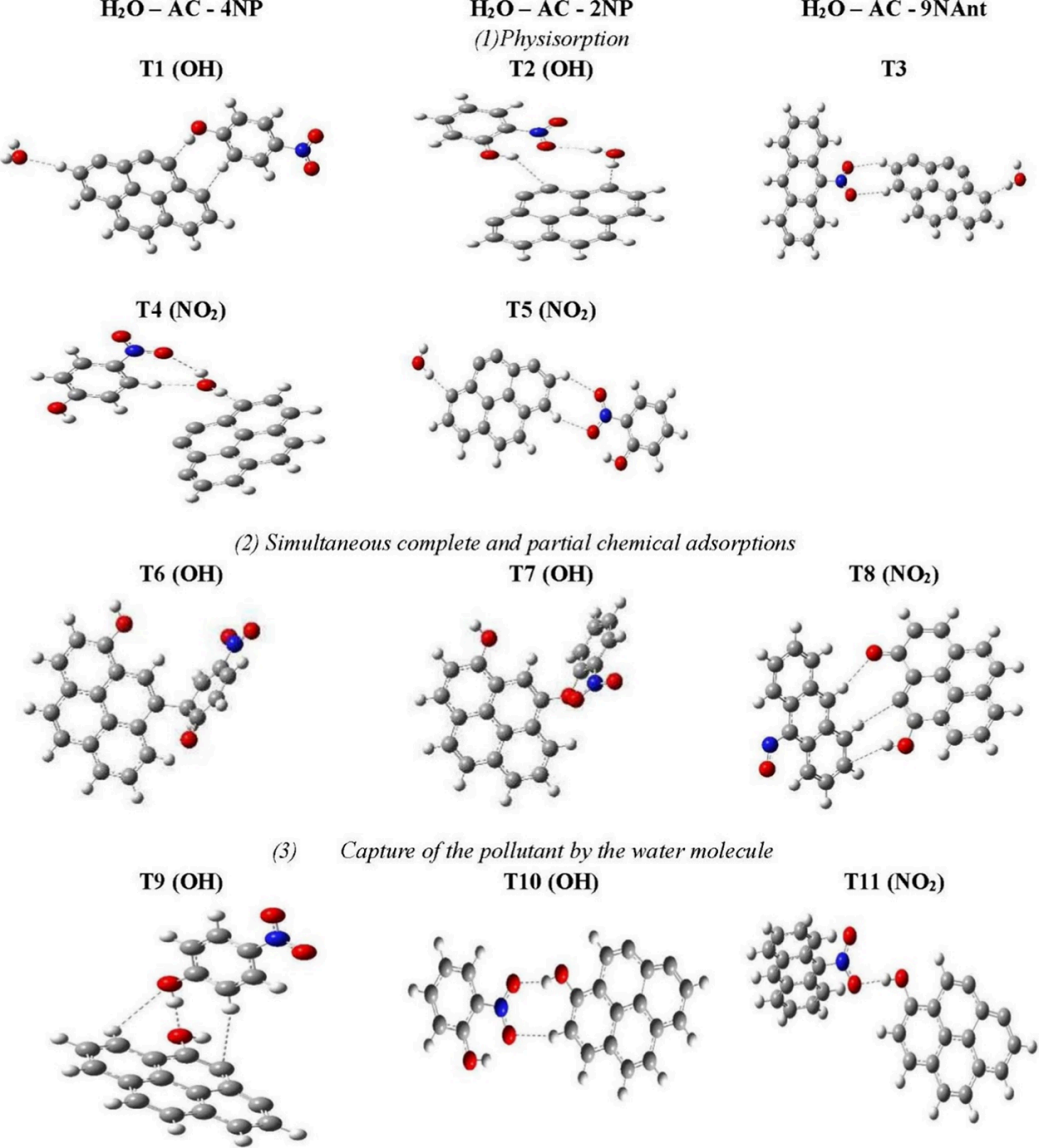
Three-body optimized structures derived from the coadsorption of one pollutant molecule and one water molecule on activated carbon. (Dotted (---) refers to the physical interactions shown in Table 3).

The most relevant configurations derived using a single water molecule are shown in Figure 4 where they are classified in three different groups: the first group (1) shows structures linked by physisorption, the second group (2) corresponds to a simultaneous chemical and physical adsorption, and the complexes of the third group (3) are produced by the capture of the pollutant by the water molecule after it is hung on AC. Optimized models of other configurations are offered in the Supporting Information. Table 3 collects structural and energetic data. TX (OH) and TX(NO2) refer to complexes linked through the OH and NO2 functional groups, respectively (X is a configuration labeling).

**Table 3:** Adsorption Energies (*E*_ads_ and *E*_adsGD3(BJ)_) Computed with and without Considering the Empirical Dispersion and Short Intermolecular Distances in the Presence of Water^a^

species		short intermolecular distances (Å)		*E*_ads_ (kJ/mol)	*E*_adsGD3(BJ)_ (kJ/mol)	BSSE (kJ/mol)
(1) *Physisorption*						
**T1** (OH)	H_2_O-AC-4NP(OH)	(AC) CH---O (H_2_O)	2.235	–25	–43	2.60
		(AC) C---HC (4NP)	2.367			
		(AC) C---HO (4NP)	2.115			
**T2** (OH)	H_2_O-AC-2NP(OH)	(AC) C---H (H_2_O)	1.962	–10	–56	2.60
		(2NP) NO_2_---H (H_2_O)	2.175			
		(AC) C---HO (2NP)	3.184			
**T3**	H_2_O-AC-9NAnt	(AC) CH---O_1_ (NO_2_-9NAnt)	2.534	–15	–93	1.57
		(AC) CH---O_2_ (NO_2_-9NAnt)	2.592			
		(AC) C---H (H_2_O)	1.926			
**T4** (NO_2_)	H_2_O-AC-4NP(NO_2_)	(AC) C---H (H_2_O)	1.939	–19	–38	2.60
		(4NP) NO_2_---H (H_2_O)	2.189			
		(4NP) CH---O (H_2_O)	2.206			
		(AC) C---H (4NP)	4.303			
**T5** (NO_2_)	H_2_O-AC-2NP(NO_2_)	(AC) C---H (H_2_O)	1.928	–13	–20	1.80
		(2NP-O_2_N) O_1_---HC (AC)	2.591			
		(2NP-O_2_N) O_2_---HC (AC)	2.593			
(2) *Simultaneous complete and partial chemical adsorptions*						
**T6** (OH)	H_2_O-AC-4NP	(AC) C–O (H_2_O)	1.369	–456	–495	10.23
		(AC) C–H (H_2_O)	1.085			
		(AC) C–CH (4NP)	1.534			
		(AC) C–HO (4NP)	1.084			
**T7** (OH)	H_2_O-AC-2NP	(AC) C–O (H_2_O)	1.371	–487	–514	11.28
		(AC) C–H (H_2_O)	1.082			
		(AC) C–O (OH–2NP)	1.386			
		(AC) C–H (OH–2NP)	1.083			
**T8** (NO_2_)	H_2_O-AC-9NAnt	(AC) C–O (H_2_O)	1.345	–300	–362	7.30
		(AC) C–H (H_2_O)	1.085			
		(AC) C–O (NO_2_-9NAnt)	1.234			
		(9NAnt) H---O (9NAnt-AC)	2.409			
		(9NAnt) H---C (AC)	3.125			
		(AC-H_2_O) OH---CH (9NAnt)	2.912			
(3) *Capture of the pollutant by the water molecule*						
**T9** (OH)	AC-H_2_O-4NP	(AC-H_2_O) HO---HO (OH-4NP)	1.851	–29	–69	2.62
		(AC) CH---OH (OH-4NP)	3.118			
		(AC) C---HC (4NP)	3.295			
		(AC) C–O (H_2_O)	1.390			
		(AC) C–H (H_2_O)	1.084			
**T10** (OH)	AC-H_2_O-2NP	(AC-H_2_O) H---O (NO_2_-2NP)	1.977	–25	–32	2.36
		(AC) CH---O (NO_2_-2NP)	2.654			
		(AC) C–O (H_2_O)	1.361			
		(AC) C–H (H_2_O)	1.086			
**T11** (NO_2_)	AC-H_2_O-9NAnt	(AC-H_2_O) H---O (NO_2_-9NAnt)	1.933	–27	–79	2.60
		(AC) C–O (H_2_O)	1.359			
		(AC) C–H (H_2_O)	1.086			

a Estimation of the superposition basis set error (BSSE) on the energy. Dotted (---) refers to the physical interactions and direct line (−) refers to a covalent bound.

The first group (1) of Figure 4 corresponds to the optimized structures resulting from the physical adsorption of pollutants on AC. Adsorption energies are less than −50 kJ/mol. The nitrophenols, 2NP and 4NP, follow opposite adsorption pathways due to the relative positions of the functional groups due to the high intrinsic hindrance of 2NP. In T1(OH), 4NP forms the three-body complex without water displacement, creating the main π–π stacking interactions between the phenolic cycle of pollutant and the AC cycles. 4NP links the AC bulk with a 2.367 Å bond. In addition, two hydrogen bonds are formed: a strong one of 2.115 Å between the OH hydrogen atom of 4NP and a C atom of the bulk and another moderately strong one of 2.235 Å between water and AC. A very similar result was observed in the formation of the two-body complex D1 (OH), which occurs from the adsorption of the same pollutant 4NP on AC without water. In this case, the adsorbed molecule and adsorbate are separated by a coherent distance of 2.365 Å. A strong hydrogen bond of 2.114 Å of the same nature has been determined.

The difference between the trimer (T1) and dimer (D1) adsorption energies is more accentuated in the presence of water. We find that the humidity plays a very important role in stabilizing the system.

On the other hand, T4(NO_2_) is produced when approached through the NO_2_ functional group. For this purpose, the water molecule varies to a new emplacement between 4NP and AC, to act as a glue between the pollutant and bulk, hindering the direct chemisorption of 4NP. A strong hydrogen bond of 1.939 Å between AC and water is created. The water performs a self-rotation to create a strong hydrogen bound of 2.189 Å, which involves a water H atom and one NO_2_ oxygen atom. The water engages its oxygen atom to produce another 2.206 Å hydrogen bond, stabilizing the system. In addition to this indirect capture of 4NP favored by H_2_O, pollutants tend to create a π–π stacking interaction between their aromatic rings and those of AC. The separation of these π–π planes is a distance of 4.303 Å, which seems a bit long because of the position of the water molecule. In 2NP, the hydroxyl group matches with the dioxide azote group and vice versa. Moreover, in T2(OH), both groups participate in the stabilization of hydrogen bonds. Indeed, the oxygen atom of the nitro group forms a strong bond of 2.175 Å with a hydrogen atom of the water molecule.

Meanwhile, the competing hydroxyl group connects its hydrogen atom to a carbon atom of AC at a distance of 3.184 Å. A strong hydrogen bond of 1.962 Å involving the water hydrogen atom and an AC carbon atom is very similar to that observed in T4(NO_2_).

T5(NO_2_) exhibits three stabilizing interactions. In two of them, the two NO2 oxygen atoms link the AC hydrogen atoms whose values are 2.591 and 2.593 Å. The last hydrogen bond of 1.928 Å, stronger than the previous bonds, occurs between the AC and water molecule. The adsorption energy is higher in 2NP due to hindrance, which makes the system less reactive than 4NP. Therefore, adsorption occurs more easily in the case of 4NP(NO_2_) compared to 2NP(NO_2_). In contrast with nitrophenol molecules, T3(9NAnt) tends to stay far away from water during the capture process due to its large surface, preventing the creation of a real link between the pollutant and AC, as occurs in nitrophenols. The pollutant interacts through its two oxygen atoms and one AC hydrogen atom. The interaction belongs to the definition limit of a hydrogen bond because the shortest interatomic distance reaches ∼2.5 Å. We also report the interaction of the water molecule with AC via a strong hydrogen bond length of 1.926 Å. Short bond distances and adsorption energies are shown in Table 3.

The second group of structures (2) are coadsorption products from V-type configurations. The high values of the adsorption energy clearly show the formation of chemical bonds. Steric effects in 2NP inhibit capture mechanisms observed in 4NP. In T6(OH), 4NP is adsorbed by forming a single ∼1.534 Å CC covalent bond and approaching benzene rings due to electron transfer. However, in T7 (OH), 2NP is adsorbed by the formation of a 1.386 Å bond between an AC carbon atom and 2NP oxygen atom. This occurs due to the adjacent emplacement of the two functional groups in 2NP and their proximity to AC. This C–O bond stabilizes the molecule, including mesomeric effects. On the other hand, concerning T8 (NO_2_), the 9NAnt capture is very weak. A cutoff of one oxygen atom from the functional group occurs and binds an AC carbon atom. The pollutant rotates to favor π–π stacking interactions between the 9NAnt rings and AC cycles, to minimize distances. Adsorption of water creates another link of 2.586 Å between a 9NAnt carbon atom and the hydrogen of the water adsorbed on AC.

In all three cases, the water is adsorbed by creating active centers capable of adsorbing a second polluting molecule.

In the third group (3), water molecules are linked to AC, via C–H and C–O bonds. The capture of pollutants occurs by the formation of strong hydrogen bonds belonging to the interval [1.85–2.91 Å] for the three trimers T9(OH), T10(OH), and T11(NO_2_), which results from the adsorption of water on the upper side of AC and the formation of a hydroxyl group, which maintains the stability of the hydrogen bonds.

If the adsorption energies of the two-body complexes (Table 2) are compared with those of the three-body complexes (Table 3), then the effect of the presence of water on *E*_ads_ can be deduced. In the case of two-body structures, *E*_ads_ values vary from −26 to −40 kJ/mol (physisorption) and from −180 to −384 kJ/mol (chemisorption). In the case of the three-body structures, *E*_ads_ varies from −10 to −25 kJ/mol (physisorption) and from −300 to −456 kJ/mol (chemisorption). In principle, it appears that water favors chemisorption but declines physisorption.

However, water molecules can act as linkers between the pollutant and bulk. This increases the range of *E*_ads_ to −25 to −27 kJ/mol (physisorption). By considering *E*_ads_ as a criterion, it can be inferred that humid environments favor the efficiency of actived carbon for the capture of nitrophenol.

Preliminary computations and tests were performed with more than one water molecule, observing that the main effect of the humidity can be described with a first single molecule, which sustains the strongest interaction with the complex. Weak additional interaction effects are obtained by adding more molecules.

**Figure 5:**
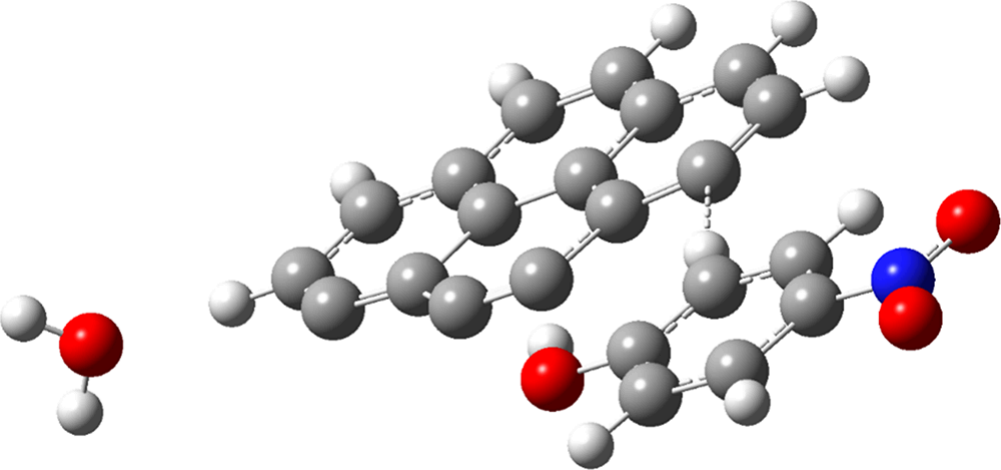
Zoomed picture of T1(OH) showing the parallel displacement of H-π-type stacking interactions.

In Figure 5, a close-up view of the T1(OH) configuration is depicted in Figure 4. This configuration demonstrates an example of π-stacking, where we observe a parallel displaced arrangement between the adjacent planes of 4NP and AC. This arrangement involves noncovalent intermolecular interactions of the H-π type, which arise from the electronic cloud of the aromatic rings present in the activated carbon. These interactions with the positively charged H group is in the −CH position of 4NP.^59,60^

In trimers, the empirical dispersion correction has no significant effect on the distances, whereas the adsorption energies seem to be too large in comparation to dimers, especially in the case of physiosorbed systems. This effect comes from the presence of water, linked to the bulk by very strong hydrogen bonds. In trimers, the BSSE correction does not exceed 13% of the energy value, except for T2, as was expected given the size of the system and the basis set used.^45^ Moreover, the BSSE correction does not change the nature of adsorption. These preliminary results are very crucial for practical modeling in the solid state.

Table 4 displays the structural parameters of AC after coadsorption. We observe that, in activated carbon (AC), symmetry breaking and molecular distortion are relatively weak. These effects are most noticeable near the intramolecular bonds and become more pronounced with an increasing number of adsorbed molecules increase.

**Table 4:** Structural Parameters of AC after the Coadsorption of 4NP, 2NP, or 9NAnt with Water on AC

	H_2_O-AC-Pollutants (a) physisorption, (b) simultaneous adsorption, (c) water adsorption								
	4-nitrophenol			2-nitrophenol			9-nitroanthracene		
parameters (Å/°)	*a*	*b*	*c*	*a*	*b*	*c*	*a*	*b*	*c*
C_17_–C_16_	1.243	1.369	1.347	1.243	1.363	1.333	1.244	1.381	1.333
C_16_–C_15_	1.415	1.431	1.404	1.415	1.442	1.404	1.414	1.449	1.404
C_5_–C_17_	1.412	1.451	1.439	1.416	1.434	1.444	1.414	1.372	1.444
C_14_–C_15_	1.373	1.411	1.353	1.372	1.404	1.369	1.387	1.396	1.370
C_5_–C_6_	1.385	1.407	1.403	1.379	1.410	1.409	1.372	1.498	1.410
C_8_–C_15_	1.468	1.424	1.512	1.468	1.429	1.498	1.463	1.431	1.498
C_5_–C_4_	1.463	1.435	1.434	1.463	1.426	1.421	1.469	1.459	1.421
C_6_–C_1_	1.386	1.394	1.395	1.381	1.397	1.399	1.367	1.475	1.399
C_13_–C_14_	1.371	1.398	1.354	1.373	1.395	1.371	1.389	1.406	1.371
C_4_–C_3_	1.429	1.432	1.435	1.431	1.429	1.435	1.431	1.406	1.435
C_8_–C_9_	1.431	1.429	1.435	1.431	1.430	1.436	1.429	1.425	1.436
C_13_–C_11_	1.371	1.393	1.402	1.399	1.394	1.397	1.393	1.386	1.397
C_1_–C_2_	1.394	1.392	1.392	1.395	1.393	1.391	1.402	1.353	1.391
C_3_–C_7_	1.429	1.439	1.433	1.431	1.437	1.431	1.431	1.425	1.431
C_9_–C_10_	1.431	1.436	1.439	1.432	1.439	1.440	1.429	1.425	1.440
C_15_C_17_C_16_	126.0	122.1	122.8	128.2	122.5	132.0	132.1	122.0	132.2
C_5_C_17_C_16_	131.7	120.3	123.5	129.6	120.0	118.0	125.6	119.4	118.0
C_17_C_5_C_6_	134.3	123.5	122.9	134.6	121.8	122.0	136.2	121.5	121.9
C_16_C_15_C_14_	135.7	122.0	138.4	135.9	122.5	137.5	133.9	124.1	137.5
C_14_C_13_C_22_	123.0	119.7	123.4	122.6	119.5	121.3	120.7	119.8	121.3
C_6_C_1_C_19_	121.1	119.4	119.0	121.5	119.6	119.6	124.4	116.3	119.6

### 3.4 Noncovalent Interaction Analysis

**Figure 6:**
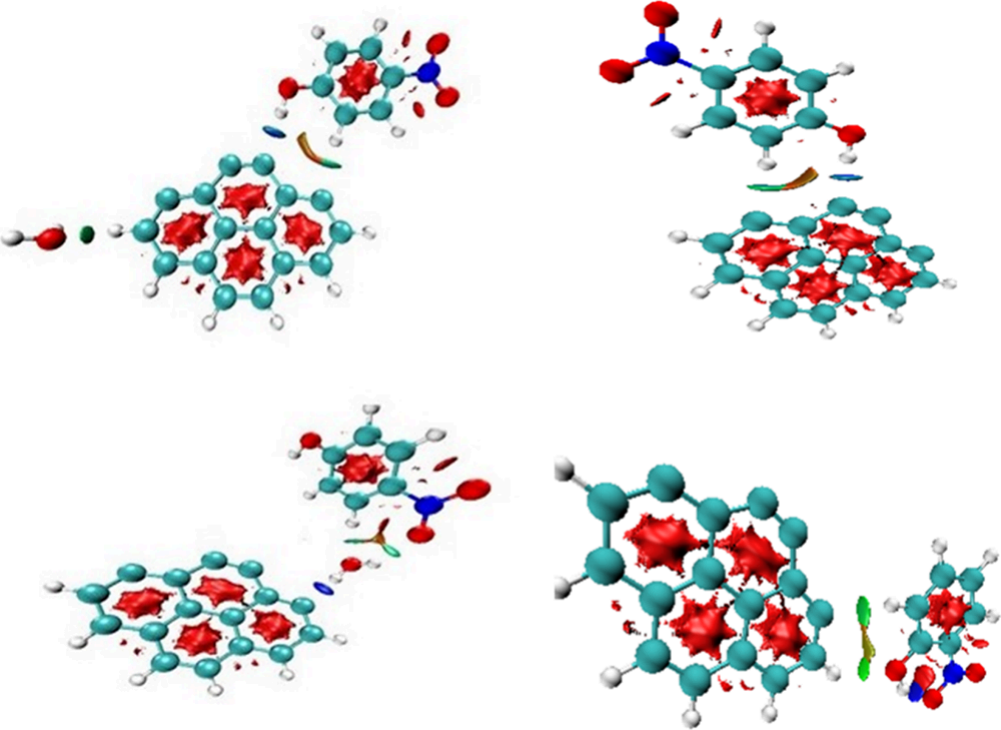
NCI analysis of the physisorbed product (T1/T4) computed at the B3LYP-GD(BJ)/6-31+G(d,p) level of theory.

To fully characterize the weak interaction observed in the previous physisorbed complex, a noncovalent interactions (NCI) analysis using the multiwfn 3.8^54^ and VMD^55^ softwares simultaneously was performed by considering different ways of physical adsorption D1, D4, T1, and T4 of nitrophenols. The results will be generalized for the other pollutants, as they have similar features. The NCI method can be viewed as an expansion of the QTAIM method, both seek to identify the nature of the interactions between atoms within molecules based on the reduced density gradient.^56,57^Figure 6 shows a blue isosurface supporting the previously observed hydrogen bound in T1 of 2.115, 1.939, and 2.114 Å in T4 (see Table 3), indicating the occurrence of hydrogen bounding. The green isosurface indicates the presence of van der Waals interactions between the phenolic ring of the pollutant or water molecule with the ring of AC, which helps to stabilize the complex. Finally, the isosurface of the AC and pollutant rings indicates a strong repulsion, shown in red. A Similar observation has been reported in previous adsorption studies involving aromatic compounds.^57,58^

### 3.5 Adsorption of More Than One Molecule of Pollutants

Simultaneous adsorptions after V approaches (see Figure 4) clearly show the formation of hydroxyl functional groups on the upper side of AC. This allows one to approach a new pollutant molecule. The results of geometry optimization form the structures of Figure 4 and are shown in Table 5. Other approaches are provided in the Supporting Information. The adsorption energy value refers to a stable physisorbed 4NP, 2NP, and 9NAnt with *E*_ads_ of −16.8, −22.1, and −25 kJ/mol, respectively, supporting the fact of the physisorbing nature of the reactivity as shown in the figures of Table 4. The intermolecular bond reached about 1.8 Å and involved the previously formed OH group. This value corresponds to a strong hydrogen-bonded interaction between an oxygen atom of one of the three pollutants and the hydrogen atom of the OH group resulting from the water-AC link. In addition to the previous interaction, 9NAnt due to its huge surface rings forms an additional π-stacking interaction between an H atom and the AC carbon atom, stabilizing the complex.

**Table 5:**
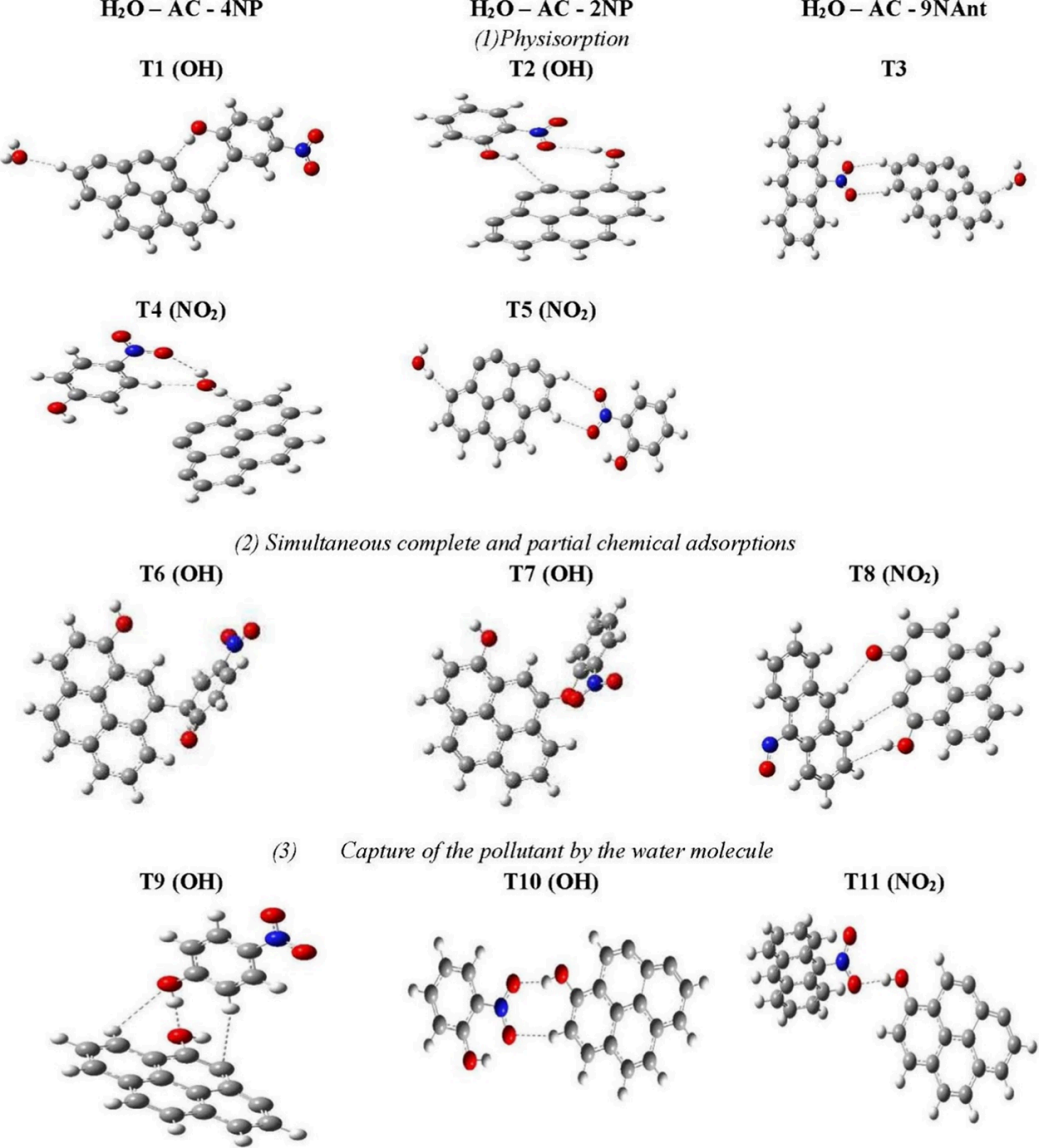
Adsorption Energies and Short Intermolecular Bond Distances between the Second Pollutant Molecule and Activated Carbon Generated from V Approaches^a^

a Dotted (---) refers to the physical interactions and direct line (−) refers to a covalent bound.

Although we conclude that water plays a very important role in favoring adsorption, we try to increase the number of water molecules. For this purpose, we swept all of the space around the adsorbate. However, the reduced C_16_H_6_ structure used to represent the AC bulk allows only one molecule of water and one pollutant to be used. For extensive work, a periodic theoretical model is needed.

### 3.6 Thermochemistry

The variation of the thermodynamic properties corresponding to the capture of 4NP, 2NP, and 9NAnt on activated carbon was calculated at the DFT/B3LYP/6-31+G(d,p) level of theory. To ensure that functional and basis changes do not impact the results, we used the same level of theory for adsorption to compare with no constraints. These properties describe well the thermodynamic stability of the complexes under the given conditions of pressure and temperature and the spontaneity of adsorption processes. Table 5 summarizes the calculated values of the standard enthalpies of formation (Δ*H*) and Gibbs free energy (Δ*G*) obtained, as follows:^44,46^
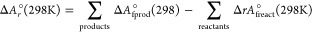
3where *A* = *H* or *G*.

The formation properties Δ*H*° and Δ*G*° of reactants (isolated molecules) and products (complexes) were taken from the Gaussian thermochemistry output, considering the zero-point vibrational energies of the corresponding optimized structures.

Analysis of the calculated thermodynamic parameters in Table 6 shows that the enthalpies are positive if the adsorption occurs through intramolecular physical interactions. In the presence of water, adsorption involves physisorption when the capture process is endothermic (Δ*H*_*r*_^°^ >0). In addition, and under normal conditions of temperature and pressure, chemical adsorption was found to be nonspontaneous (Δ*G*_*r*_^°^ >0). This outcome is in good agreement with previous results found in literature.^47−53^ Indeed, the thermodynamic parameters of adsorption of 4-nitrophenol (4NP) on poly(vinyl alcohol)/activated carbon^48^ and other organic molecules on activated carbon have been studied experimentally. The value of (4NP) on poly(vinyl alcohol) on activated carbon are found to be 1.91 kJ/mol for Δ*G*_*r*_^°^ and 12.88 kJ/mol, revealing physical, endothermic, and nonspontaneous processes.^48,53^

**Table 6:** Thermodynamic Properties of the Different Complexes Were Computed at the DFT/B3LYP/6-31+G(d,p) Level of Theory

		thermodynamic properties(kJ/mol)			
		298 K		1000 K	
compound	complexes	Δ*H*	Δ*G*	Δ*H*	Δ*G*
**D1**	AC-4NP (OH)	5.25	2.36	1.84	3.15
**D2**	AC-4NP (NO_2_)	–343.94	–287.75	–339.47	–152.54
**D3**	AC-2NP (OH)	–134.95	–78.50	–142.82	31.27
**D4**	AC-2NP (NO_2_)	12.33	8.14	0.26	0.52
**D5**	9NAnt (NO_2_)-AC	–347.09	–286.17	–346.30	–136.5
**D6**	AC-H_2_O	–377.31	–77.184	–379.91	–213.45
(1) *Physisorption*					
**T1**	H_2_O–AC-4NP (OH)	7.35	11.80	1.84	–1.57
**T2**	H_2_O–AC-2NP (OH)	16.50	21.00	1.31	–1.56
**T3**	H_2_O–AC-9NAnt	7.61	14.4	4.72	4.98
**T4**	H_2_O-AC-4NP (NO_2_)	6.30	12.30	2.62	1.57
**T5**	H_2_O–AC-2NP (NO_2_)	8.40	12.86	4.98	2.886
(2) *Simultaneous complete and partial chemical adsorptions*					
**T6**	H_2_O-AC-4NP (OH)	–818.36	–707.56	–816.00	–470.48
**T7**	H_2_O-AC-2NP (OH)	–840.41	–721.48	–844.36	–509.87
**T8**	H_2_O-AC-9NAnt	–658.73	–599.39	–662.15	–481.77
(3) *Capture of the pollutant by the water molecule*					
**T9**	AC-H_2_O-4NP (OH)	–384.63	–326.61	–386.47	–212.13
**T10**	AC-H_2_O-2NP (OH)	–369.93	–309.54	–379.12	–209.51
**T11**	AC-H_2_O-9NAnt (NO_2_)	–378.07	–319.78	–379.12	–206.36

Therefore, physisorption is favored thermodynamically but requires heat to occur. However, the positive value of Δ*H*_*r*_^°^ indicates an endothermic process, which means that, in these configurations, the capture of pollutants is eco-friendly because it does not delegate gas.

On the other hand, in cases where adsorption involves the formation of new bonds between the pollutant and activated carbon, Δ*G*_*r*_^°^ and Δ*H*_*r*_^°^ are negative and very high, giving rise to an exothermic process. For this purpose, chemical adsorption is disadvantaged from a thermodynamic point of view, since it releases heat to the environment.

Concerning the conformations of groups 2 and 3, the change in the enthalpies (Δ*H*_*r*_^°^) and the change in Gibbs free energies (Δ*G*_*r*_^°^) are negative, indicating that the adsorption takes place via spontaneous and exothermic phenomenon and chemisorption. These values became more negative when we move from group 3 to group 2, which is due to the total chemisorption in the last group. Meanwhile, it is partial in the conformations of group 3.

The adsorption of 2NP through the OH functional group to form the two-body complex D3 is exothermic and spontaneous and reveals chemisorption at 298 K (Δ*H* = −134.9 kJ/mol; Δ*G* = −78.50 kJ/mol). However, if the pollutant and water are coadsorbed to form the T2 trimer, the process is endothermic and nonspontaneous and occurs toward physisorption ((Δ*H* = 16.50 kJ/mol; Δ*G* = 21.0 kJ/mol). Indeed, in T2, the emplacement of water between the pollutant and the bulk prevents the formation of chemical bonds. However, the formation of T2 occurs spontaneously (Δ*G* = −1.56 kJ/mol) by increasing the temperature to 1000 K.

Adsorption of 4NP through OH and NO_2_ groups in the presence of water yields similar thermodynamic properties at 298 K. However, at 1000K, this adsorption through OH becomes spontaneous. At room temperature and without water, the 2NP and 4NP show opposite behaviors. Adsorption through OH is spontaneous for 2NP, while adsorption through NO_2_ is spontaneous for 4NP.

On the other hand, at room temperature, the dry adsorption of 9NAnt on activated carbon produces D5 following an exothermic and spontaneous process and giving rise to chemisorption (Δ*G* = 347.09 kJ/mol). When partially adsorbed in the presence of water (T3), both Δ*H* and Δ*G* are positive. At 298 K, adsorption becomes endothermic, nonspontaneous and of physical nature in a humid environment. However, if the temperature increases, the free energy decreases, indicating a tendency toward spontaneity.

Generally, all endothermic processes exhibit positive Gibbs free energy values, which means that they are not spontaneous. The addition of the water molecule following a physisorption process decreases Δ*G* and tends toward spontaneity.

By comparing the two pollutants 2NP and 4NP, it can be inferred from the computed thermodynamic properties that the capture is influenced by the steric hindrance of the adsorbed molecules and the distribution in their structure of the hydroxyl and nitrogen dioxide groups.

It is noticeable that the enthalpies decrease when the temperature increases as was expected because endothermic processes favor high temperatures. This effect is very pronounced in the presence of the water molecule.

## 4 Conclusions

This paper represents a systematic investigation at the molecular level of the adsorption of 4NP, 2NP, and 9NAnt molecules on activated carbon in the gas phase under dry and humid conditions. Density functional theory calculations reveal favorable adsorption for different species on the upper unsaturated side of AC. Adsorption can be stimulated by humidity.

Optimized structures of isolated systems show various pathways for the capture. Water can act as a linker between the bulk and adsorbate but can generate competition, because given its polar behavior, it can be linked directly to the nonpolar surface of AC.

On the other hand, the simultaneous adsorption of water and pollutants occurs when the two adsorbates approach the bulk following opposite directions. In these cases, the adsorption energy is negative (−456, −487, and −300 kJ/mol for 4NP, linked to the bulk in a simultaneous adsorption process; AC shows a remarkable ability for the capture of a second pollutant molecule, due to the strong hydrogen bond. This achieves significant stability of the final systems. From the preliminary tests performed with more than one water molecule, we can conclude that the main effect and strong interaction occur mainly with the first water and main initial complex.

The thermodynamic study indicates that the physisorption through van der Waals interactions, hydrogen bonds, or π–π-stacking interactions represents an eco-friendly pathway. The presence of water decreases the free Gibbs energy and increases the spontaneity of these reactions, although a high temperature is needed to obtain a positive value of Δ*G*.

The addition of nitrophenol initiated by a V configuration is not suggested because this approach carries out an exothermic value due to the creation of a real link between AC and the pollutants. The addition of a water molecule has a significant effect on the thermodynamic parameters favoring physisorption and revealing an endothermic and nonspontaneous process, which tends toward spontaneity or becomes totally spontaneous, at high temperature.

It is clear that all the different approaches can lead to stable adsorption either with the fusion of pollutants on AC by creation of new bonds or by physical interaction like hydrogen bonds, π–π-stacking, electron transfer, or hydrophobic interactions. In addition, water can be beneficial for the capture of the three studied pollutants because humid conditions interfere between the pollutant and AC, making the reaction endothermic, which means eco-friendly for the environment.

In any case, it can be concluded that the chemical approaches applied in this study of the adsorption of pollutants on AC in the presence of water represent a first description of behavior endorsed by previous studies, but they can be completed in further studies using solid-state modeling.

## Supporting Information Available

Python scripts, optimized structures with different approaches of adsorption process, and the Cartesian coordinates (PDF)
