# Supplementary material for: The Role of Water in the Adsorption of Nitro-Organic Pollutants on Activated Carbon
Source: J Phys Chem A. 2023 Sep 25;127(39):8146–58. doi: 10.1021/acs.jpca.3c03877 (PMC10561263; doi:10.1021/acs.jpca.3c03877)
Supplement: Supplementary file 1 — jp3c03877_si_001.pdf [file jp3c03877_si_001.pdf]

Supporting Information for:

The Role of Water in the Adsorption of Nitro Organics Pollutants on Activated Carbon

Celia Adjal[a,b], Vicente Timón[b], Nabila Guechtouli[a,c], Rahma Boussassi[a], Dalila Hammoutène[a], and María Luisa Senent[b,*].

a) USTHB, Laboratory of Thermodynamics and Molecular Modelling, Faculty of Chemistry, BP32, El Alia, 16111, Bab Ezzouar, Algiers, Algeria.
b) Instituto de Estructura de la Materia, CSIC, Serrano 121, Madrid 28006, SPAIN.
c) University of Mouloud Mammeri of Tizi Ouzou, UMMTO, Faculty of Sciences, Departement of Chemistry
* Corresponding author; E_mail: ml.senent@csic.es

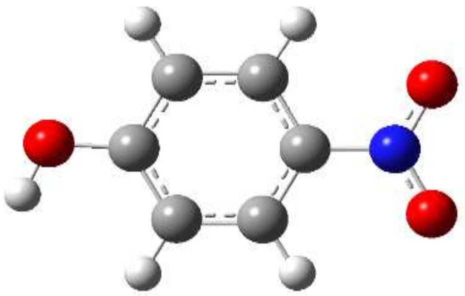

4Nitrophenol (4NP)

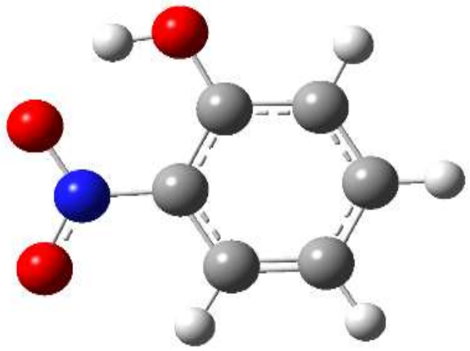

2Nitrophenol (2NP)

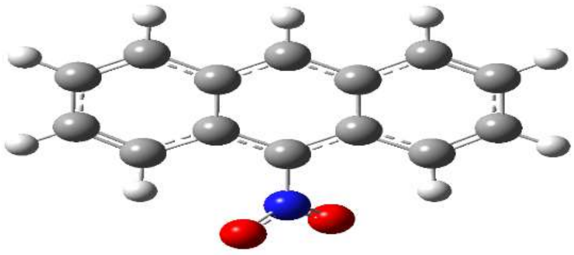

9Nitroanthracene (9NAnt)

Figure S1. Optimized structures of pollutants

Table S1

Different approaches and optimized geometry of 4NP and water onto AC

Configurations	Initial geometry	Optimised geometry
	4NP (OH side) fix	
A1	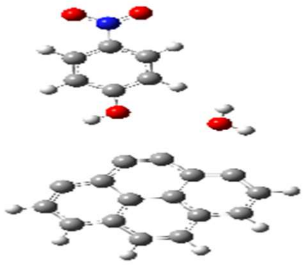	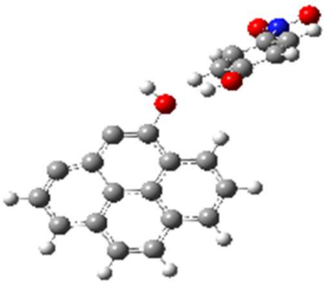
B1	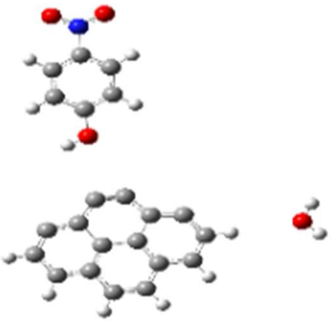	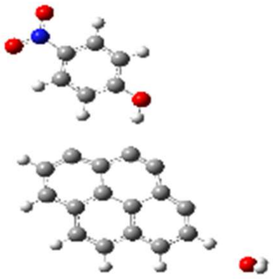
C1	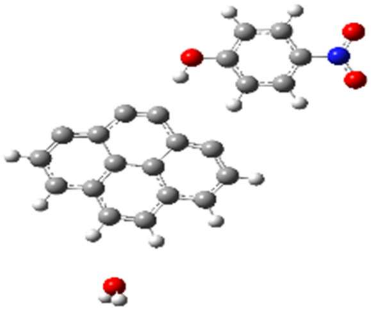	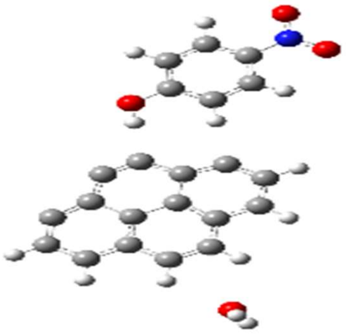
4NP (Nitro side) fix		

A3	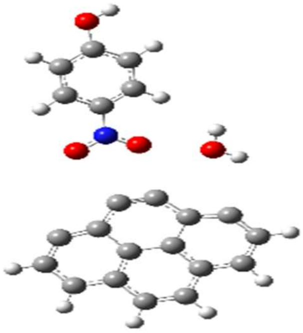	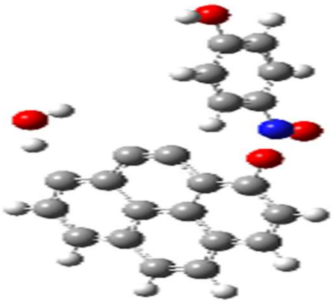
B3	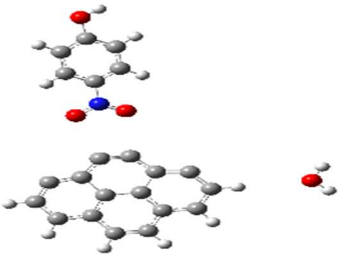	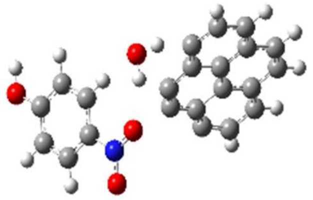
C3	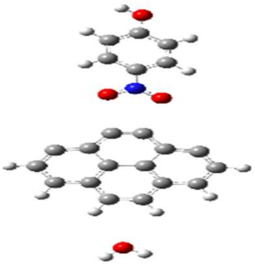	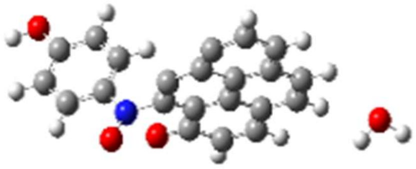
H_2O fix / 4NP-OH side		
A2	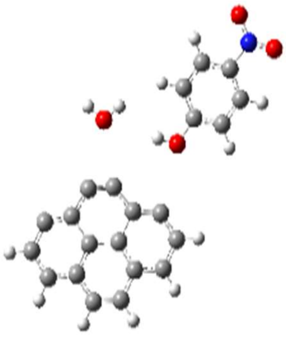	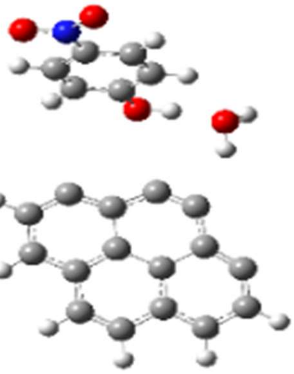

B2	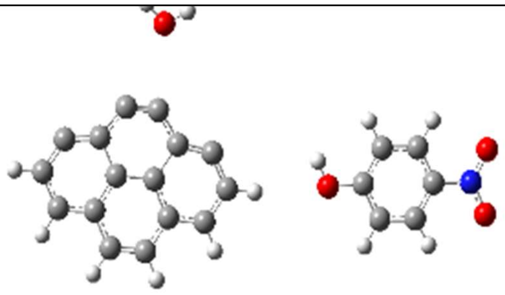	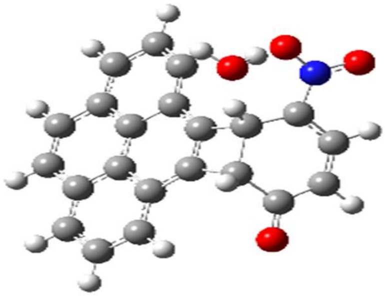
C2	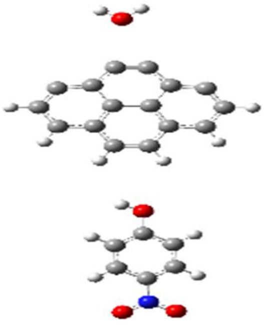	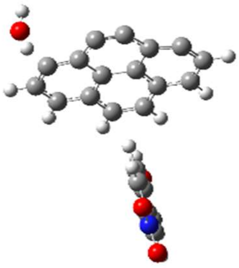
H_2O fix / 4NP-Nitro side		
A4	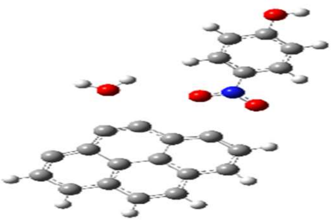	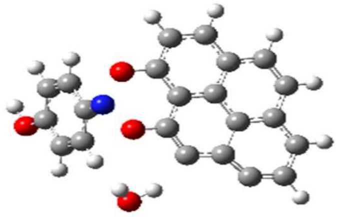
B4	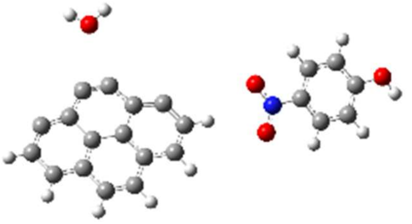	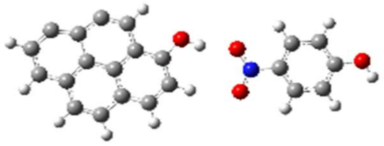

C4	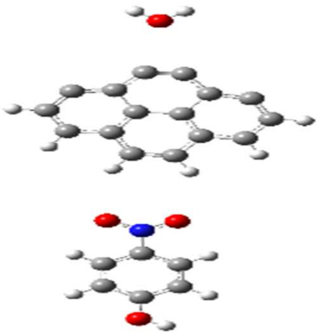	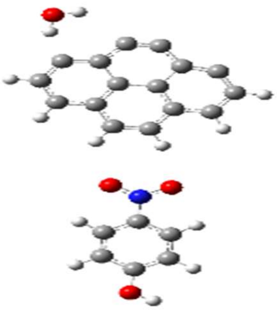
V approaches		
D1	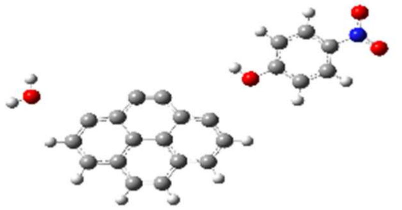	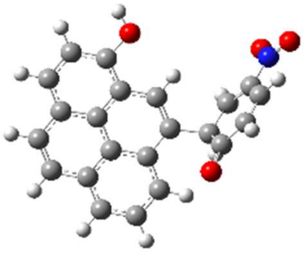
D2	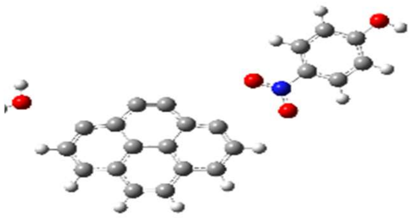	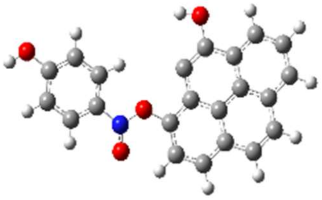
Perpendicularly approaches		
E1	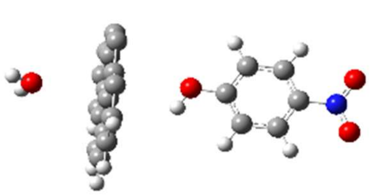	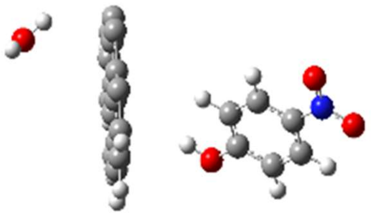

E2	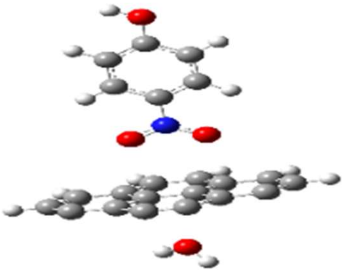	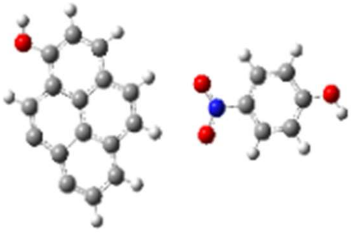
Addition of one 4NP		
C	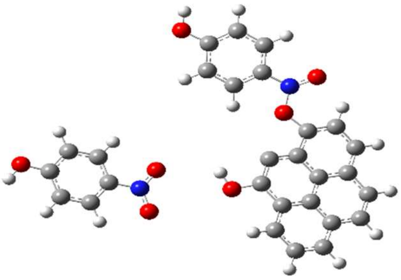	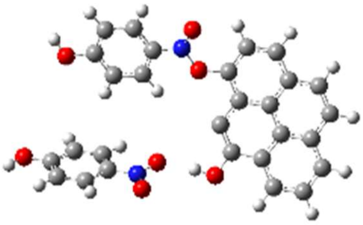

Table S2 Different approaches and optimized geometry of 2NP and water onto AC

Configurations	Initial geometry	Optimised geometry
	2NP (Nitro side) fix	
A1	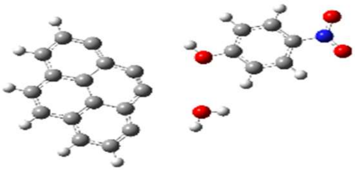	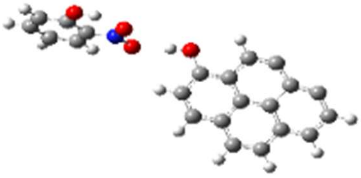
B1	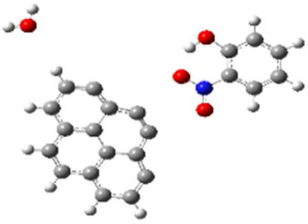	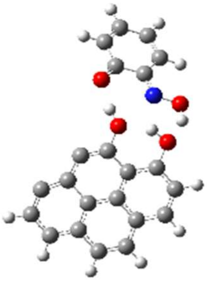

C1	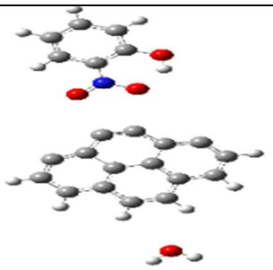	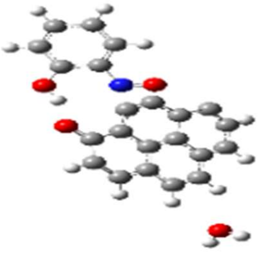
2NP (OH side) fix		
A2	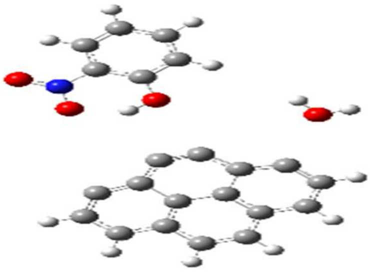	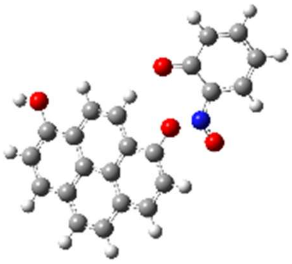
B2	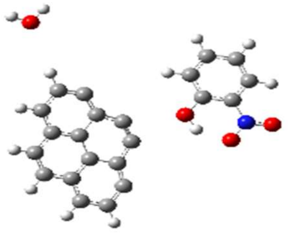	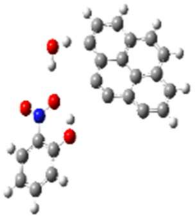
C2	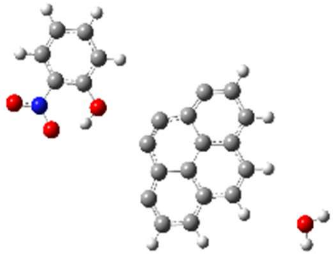	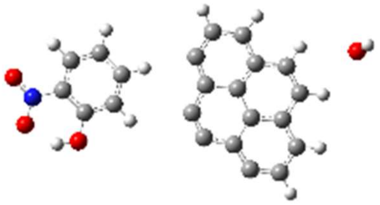
H_2O fix / 2NP-OH side		

A3	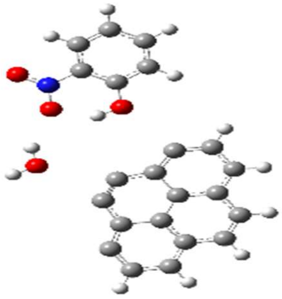	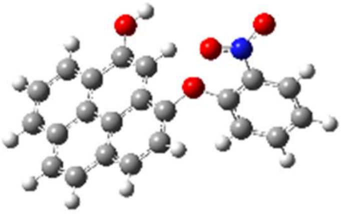
B3	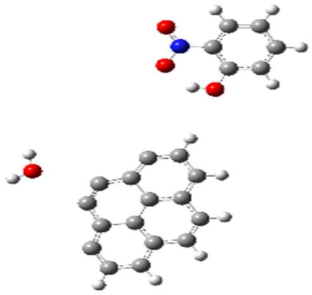	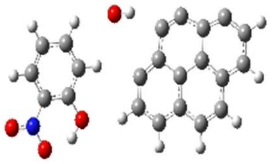
C3	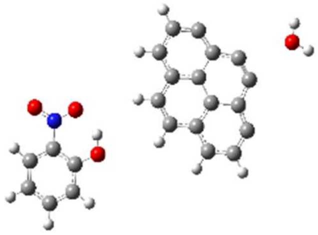	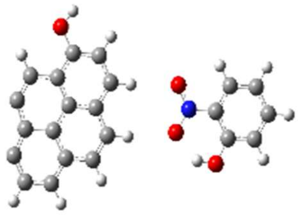
H_2O fix / 2NP-Nitro side		
A4	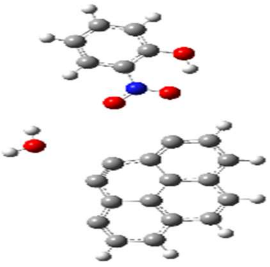	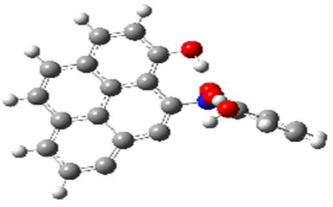

B4	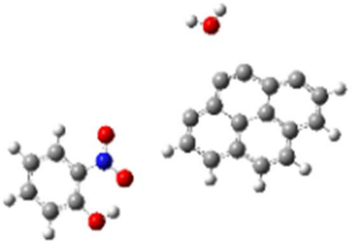	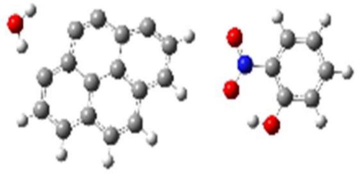
C4	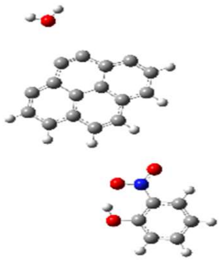	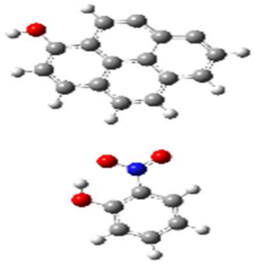
V approaches		
D1	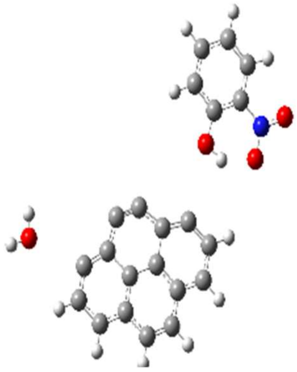	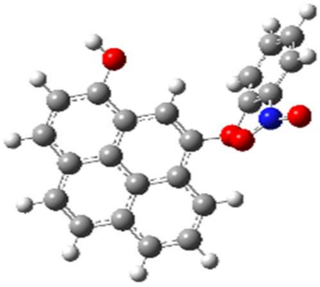
D2	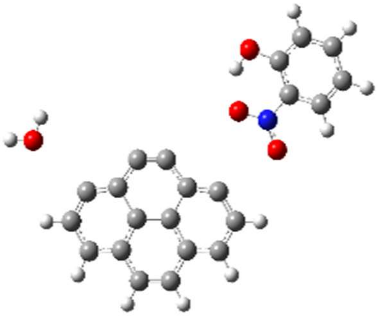	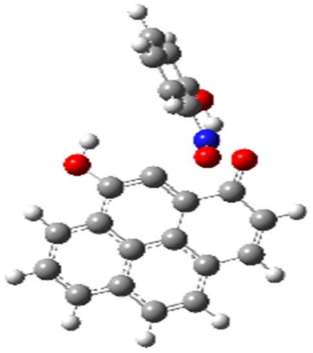
Perpendicularly approaches		

E1	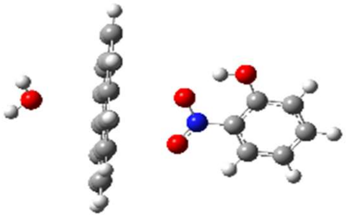	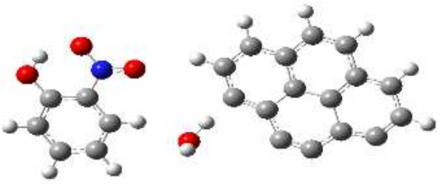
E2	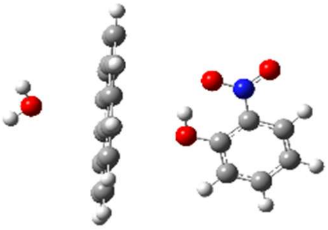	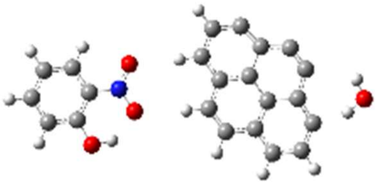
Add of one 2NP		
C	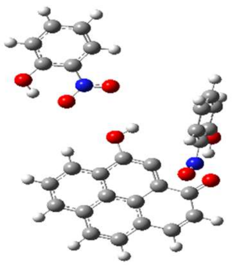	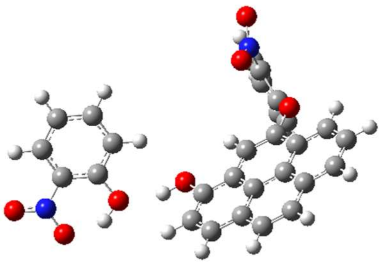

Table S3 Different approaches and optimized geometry of 9NAnt and water onto AC

Configurations	Initial geometry	Optimised geometry
	9NAnt fix	
A1	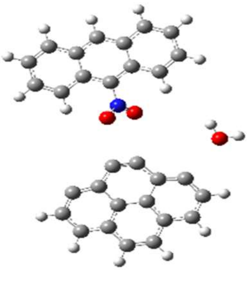	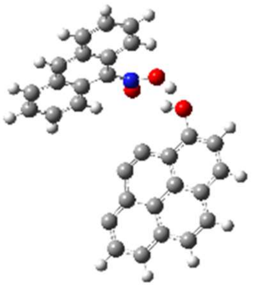

B1	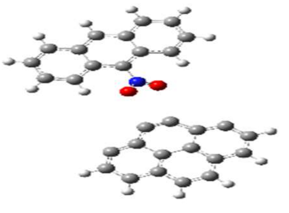	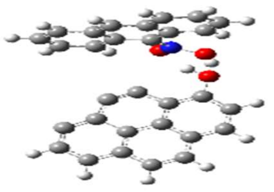
C1	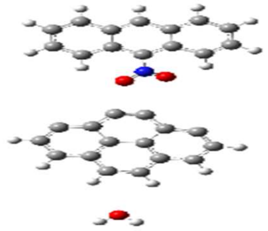	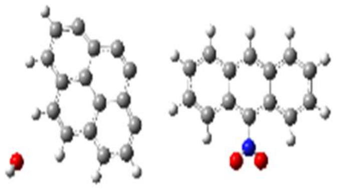
	H_2O fix	
A2	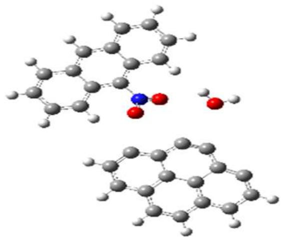	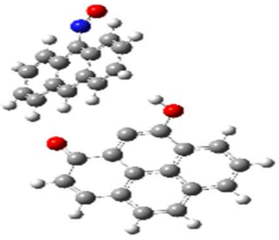
B2	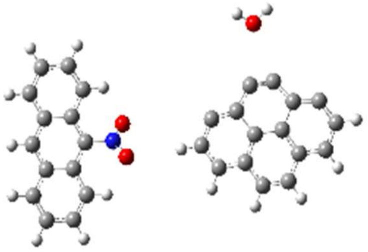	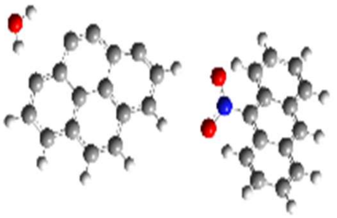
C2	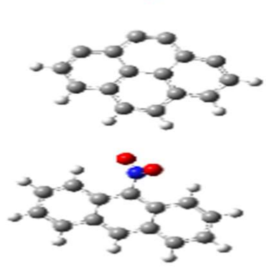	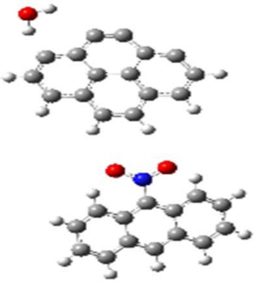

V approach		
D	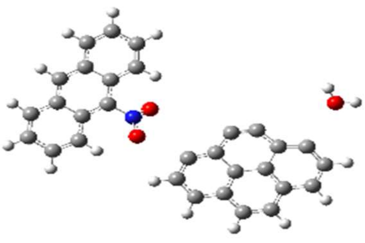	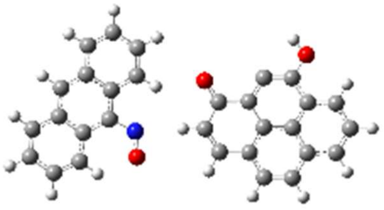
Perpendicularly approaches		
E	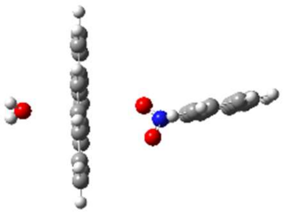	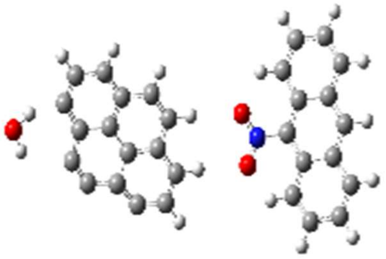

Table S4 Cartesian cordinates of optimized parameters

	Cartesian coordinates
AC	0 1<br>C 0.00000000 3.57702500 -0.06656600<br>C 0.00000000 2.83696000 1.12274900<br>C 0.00000000 1.42819900 1.08492700<br>C 0.00000000 0.73346500 -0.16761900<br>C 0.00000000 1.50993300 -1.41374100<br>C 0.00000000 2.86335000 -1.23493900<br>C 0.00000000 0.67904500 2.31229200<br>C 0.00000000 -0.73346500 -0.16761900<br>C 0.00000000 -1.42819900 1.08492700<br>C 0.00000000 -0.67904500 2.31229200<br>C 0.00000000 -2.83696000 1.12274900<br>H 0.00000000 -3.34901400 2.08280000<br>C 0.00000000 -3.57702500 -0.06656600<br>C 0.00000000 -2.86335000 -1.23493900<br>C 0.00000000 -1.50993300 -1.41374100<br>C 0.00000000 -0.62039500 -2.51854200<br>C 0.00000000 0.62039500 -2.51854200<br>H 0.00000000 1.22967700 3.24853900

	H 0.00000000 4.66414500 -0.04270300<br>H 0.00000000 3.34901400 2.08280000<br>H 0.00000000 -1.22967700 3.24853900<br>H 0.00000000 -4.66414500 -0.04270300
4NP	0 1<br>C 0.01287600 1.22335500 0.00017800<br>C 0.69193900 0.00069000 0.00000100<br>C 0.00510300 -1.21427200 -0.00021200<br>C -1.38574200 -1.20583400 -0.00018200<br>C -2.07777600 0.01475400 0.00009800<br>C -1.37528800 1.22937200 0.00024700<br>H 0.57757600 2.14744100 0.00028900<br>H 0.56088500 -2.14374900 -0.00035800<br>H -1.93338900 -2.14485000 -0.00036400<br>H -1.93193500 2.15997000 0.00038400<br>O -3.43623900 0.08682200 0.00001900<br>H -3.82492800 -0.79849000 -0.00069500<br>N 2.15558200 -0.00673900 -0.00004500<br>O 2.73810800 1.08102600 -0.00054000<br>O 2.72763600 -1.10054000 0.00055700
2NP	0 1<br>C 1.90940400 -1.41365700 -0.00027900<br>C 2.55806200 -0.16431200 -0.00000900<br>C 1.83318700 1.01721400 0.00019800<br>C 0.42630000 0.99618700 0.00012600<br>C -0.21665200 -0.26824600 -0.00003300<br>C 0.52662500 -1.46164300 -0.00023200<br>H 2.48524000 -2.33257900 -0.00042500<br>H 3.64317500 -0.12054200 -0.00019100<br>H 2.32179900 1.98548700 0.00020800<br>H -0.01080700 -2.40193300 -0.00040000<br>O -0.21709200 2.16986600 0.00042100<br>H -1.18331000 1.97131400 -0.00017700<br>N -1.66031900 -0.36368200 0.00001000<br>O -2.19102000 -1.47022800 0.00086200<br>O -2.32381500 0.70170800 -0.00099700
9NAnt	0 1<br>O -0.57667600 2.82563100 -0.92578900<br>O 0.57630900 2.82579100 0.92578900<br>N -0.00014200 2.25184200 0.00002300<br>C -1.24383500 0.11747000 -0.00325200<br>C 1.24387200 0.11755300 0.00330000<br>C -1.22526800 -1.32783800 0.01245800<br>C 1.22538800 -1.32776500 -0.01240400<br>C -0.00001500 0.77790300 0.00005000<br>C 0.00008100 -2.00379000 0.00004100<br>C -2.51274400 0.78023100 0.01898300<br>C 2.51276800 0.78036400 -0.01904900<br>C -2.46727700 -2.03760600 0.04130900

	C 2.46743200 -2.03747700 -0.04128800<br>C -3.67825900 0.05721700 0.05203900<br>C 3.67831100 0.05739800 -0.05213500<br>C -3.66186100 -1.36761900 0.06061300<br>C 3.66198000 -1.36743800 -0.06065100<br>H 0.00011100 -3.09115900 0.00003500<br>H -2.55190000 1.86223700 -0.00294500<br>H 2.55190000 1.86236500 0.00283900<br>H -2.43944400 -3.12375300 0.05126900<br>H 2.43965000 -3.12362500 -0.05123000<br>H -4.62991900 0.57977500 0.06906900<br>H 4.62995000 0.57999500 -0.06922600<br>H -4.59857800 -1.91596900 0.08371600<br>H 4.59872200 -1.91574500 -0.08378300
D1	0 1<br>C -5.69317100 -2.26175700 -0.01284400<br>C -6.00173400 -0.89577700 -0.00772100<br>C -4.97270000 0.06731000 -0.00396400<br>C -3.59733200 -0.33208000 -0.00482600<br>C -3.27516900 -1.76859700 -0.00962500<br>C -4.36411500 -2.58470100 -0.01849400<br>C -5.30423800 1.46644000 0.00252400<br>C -2.55683600 0.69898600 0.00123500<br>C -2.94484800 2.07795900 0.00756100<br>C -4.34013700 2.42291000 0.00799900<br>C -1.96989900 3.09652800 0.01495200<br>H -2.28081500 4.13889100 0.01863500<br>C -0.60662100 2.77443200 0.01636200<br>C -0.30680800 1.43931200 0.00480700<br>C -1.13371200 0.36051000 0.00302100<br>C -0.93762900 -1.06261700 -0.00062200<br>C -1.87021800 -1.89053900 -0.00639700<br>H -6.35425100 1.74319400 0.00286400<br>H -6.48069100 -3.01094700 -0.01668000<br>H -7.04039500 -0.57300700 -0.00796800<br>H -4.60869300 3.47505900 0.01277100<br>H 0.15079100 3.55391800 0.02184300<br>C 5.25241300 -1.41515100 0.01374600<br>C 5.09018600 -0.02514800 -0.00410600<br>C 3.81938000 0.55490300 -0.01371100<br>C 2.69606100 -0.26231400 -0.00551500<br>C 2.84299800 -1.66101100 0.01250500<br>C 4.12993400 -2.22971500 0.02198700<br>H 6.25039700 -1.83578000 0.02092100<br>H 3.72564700 1.63376500 -0.02761400<br>H 1.70260900 0.19107400 -0.01297200<br>H 4.22251700 -3.31019400 0.03584900<br>O 1.79507200 -2.51226200 0.02155400<br>H 0.94014700 -2.03480700 0.01341500

	N 6.26847200 0.83571100 -0.01271500<br>O 7.38054600 0.29773200 -0.00410200<br>O 6.09420800 2.05947500 -0.02811500
D2	0 1<br>C 4.61696000 -2.22220600 0.58130000<br>C 4.84153000 -0.89447700 0.93949900<br>C 3.83945900 0.08782800 0.73414100<br>C 2.57115700 -0.30702700 0.20744300<br>C 2.27064200 -1.73019100 -0.01162800<br>C 3.31779600 -2.62306800 0.32807700<br>C 4.13365300 1.46953500 0.88603400<br>C 1.63936700 0.69274700 -0.21047200<br>C 1.99619800 2.07321700 -0.10204700<br>C 3.23494500 2.43115600 0.46866800<br>C 1.08490200 3.06251800 -0.60940500<br>H 1.37434300 4.10786200 -0.52670100<br>C -0.07550400 2.72619600 -1.23741700<br>C -0.49392400 1.33648200 -1.38924200<br>C 0.36819300 0.33429900 -0.73892000<br>C 0.00986500 -1.04110700 -0.65915400<br>C 0.96124500 -2.02416200 -0.40528700<br>H 5.10418400 1.75935900 1.27788700<br>H 5.41878800 -2.95254600 0.66692100<br>H 5.78907800 -0.59838900 1.38551400<br>H 3.48422200 3.48479200 0.56245500<br>H -0.72905600 3.47638000 -1.67113200<br>C -2.23452500 -0.14096000 1.07024800<br>C -2.42158800 -0.86372200 -0.11974500<br>C -3.70654900 -1.05986000 -0.64385200<br>C -4.80378700 -0.52802700 0.01556500<br>C -4.62063300 0.19655900 1.20460300<br>C -3.33319600 0.38184300 1.73301600<br>H -1.24162300 -0.01209400 1.48362700<br>H -3.82120100 -1.60705900 -1.57141600<br>H -5.80112400 -0.65896500 -0.39628600<br>H -3.21847100 0.92870000 2.66219100<br>O -5.65484200 0.74428000 1.89386900<br>H -6.49484600 0.56325400 1.45007600<br>N -1.32787800 -1.48938700 -0.77427800<br>O -1.53277100 1.01980100 -2.00158200<br>O -1.53068500 -2.54045800 -1.41125500
D3	0 1<br>C -4.59561800 -2.65293200 -0.33864700<br>C -5.04730900 -1.34696100 -0.13007000<br>C -4.14144200 -0.27312600 -0.03175800<br>C -2.73801800 -0.51569300 -0.14600600<br>C -2.29817300 -1.90716600 -0.37804700

	C -3.23901200 -2.91206500 -0.48603900<br>C -4.59932200 1.07154400 0.18884700<br>C -1.82040300 0.58845200 -0.02989700<br>C -2.31485100 1.91500300 0.18502900<br>C -3.73215500 2.11769700 0.29280400<br>C -1.40277700 2.98666100 0.28679100<br>H -1.78590600 3.98987800 0.44861900<br>C -0.02870100 2.78625600 0.19146000<br>C 0.45089000 1.49584900 -0.01130300<br>C -0.39197600 0.39052200 -0.13402700<br>C -0.02561500 -0.96622900 -0.35832200<br>C -0.88604300 -1.85870600 -0.45188300<br>H -5.66986200 1.23934900 0.27146400<br>H -5.32505100 -3.45917100 -0.39942400<br>H -6.11343300 -1.14518400 -0.04318800<br>H -4.10096200 3.12632800 0.45724900<br>H 0.66953400 3.61322800 0.26906200<br>C 4.03882100 -1.49686800 1.97241500<br>C 2.95623200 -0.83114200 2.57742500<br>C 2.20326700 0.08940000 1.85666600<br>C 2.50955700 0.35957900 0.52345200<br>C 3.61400500 -0.29647100 -0.09169100<br>C 4.36823900 -1.23606200 0.65779600<br>H 4.61909700 -2.21897100 2.53664300<br>H 2.70199800 -1.03265300 3.61275600<br>H 1.36422400 0.60327800 2.31156500<br>H 5.19531500 -1.73308700 0.16724900<br>O 1.84254600 1.32847500 -0.19343500<br>H 2.57458300 1.15352700 -1.65116500<br>N 3.99580000 -0.09240400 -1.41625900<br>O 4.94173100 -0.66303400 -1.95449600<br>O 3.29350900 0.76769100 -2.21919100
D4	0 1<br>C 0.16592200 1.35941900 -0.09687800<br>C 1.27750700 2.20110800 0.02568500<br>C 2.58187000 1.66479200 0.06271000<br>C 2.78781900 0.25079200 -0.02570100<br>C 1.62018900 -0.62817300 -0.15554700<br>C 0.40942800 0.00862600 -0.11308700<br>C 3.71499800 2.54114900 0.16647800<br>C 4.15675200 -0.27060700 -0.00746500<br>C 5.25025500 0.64813900 0.09764500<br>C 4.98579400 2.05744800 0.18313900<br>C 6.58141400 0.18107700 0.09521100<br>H 7.39996200 0.89168600 0.18912000<br>C 6.85224700 -1.18925100 -0.00719100<br>C 5.76769200 -2.02479900 -0.03682300<br>C 4.44141200 -1.70732900 -0.11665500<br>C 3.22196200 -2.41831700 -0.24587800

	C 2.06395600 -1.97103200 -0.26238600<br>H 3.53242700 3.60979500 0.23219800<br>H -0.84304400 1.76472300 -0.12058700<br>H 1.13901400 3.27738500 0.10562100<br>H 5.83139500 2.73441300 0.26246400<br>H 7.87629100 -1.55433400 -0.00278800<br>C -4.99863000 -2.57367100 0.24039000<br>C -3.60886600 -2.34988500 0.21746700<br>C -3.09080000 -1.06918400 0.10123500<br>C -3.94831000 0.04072900 0.00281300<br>C -5.34712400 -0.19443900 0.02676400<br>C -5.86256100 -1.49747600 0.14506600<br>H -5.39220300 -3.58011000 0.33171400<br>H -2.92551300 -3.19061400 0.29177900<br>H -2.01815200 -0.89002300 0.08358600<br>H -6.93780000 -1.62631700 0.15869100<br>O -3.39584700 1.25894400 -0.10795800<br>H -4.14054300 1.90511800 -0.16631500<br>N -6.28377100 0.90265400 -0.07053200<br>O -7.48884000 0.67193500 -0.04701400<br>O -5.82443800 2.06724800 -0.17674100
D5	0 1<br>C 2.82757500 -3.08675300 0.57450900<br>C 4.09276500 -2.53840800 0.31226600<br>C 4.28055300 -1.16837800 0.02317700<br>C 3.13482300 -0.31385200 0.01246000<br>C 1.90146200 -0.90090300 0.29718000<br>C 1.70835600 -2.25098200 0.56373600<br>C 5.55278900 -0.57007800 -0.26177400<br>C 3.20732700 1.08380100 -0.26803600<br>C 4.49358000 1.63671700 -0.56155700<br>C 5.64373800 0.76236300 -0.53863800<br>C 4.62007300 2.99614700 -0.87759500<br>H 5.59446000 3.42532200 -1.09483900<br>C 3.46651700 3.78841100 -0.91048900<br>C 2.27551200 3.19464700 -0.59381600<br>C 1.95353500 1.90630400 -0.26459200<br>C 0.78825300 1.17488400 0.07574700<br>C 0.69939700 -0.12659200 0.36558800<br>H 6.44447500 -1.19095600 -0.25714400<br>H 2.73813600 -4.15023000 0.78128300<br>H 4.96413800 -3.18785300 0.32539300<br>H 6.61137900 1.20481800 -0.75800000<br>H 3.52144200 4.84393100 -1.16522800<br>O -2.02463100 0.50270600 2.90302200<br>O -2.12276000 2.25875200 1.61448000<br>N -2.21356300 1.05001500 1.81366500<br>C -1.77286000 -0.83810600 0.28300400

	C -3.86569700 0.48299400 0.05674200<br>C -2.20067300 -1.66709200 -0.80451000<br>C -4.25854900 -0.35787500 -1.03452000<br>C -2.60941500 0.18972200 0.67389500<br>C -3.40410600 -1.41291600 -1.43552200<br>C -0.40335600 -1.06563300 0.89669400<br>C -4.74678400 1.51785500 0.47299600<br>C -1.38371800 -2.81372900 -1.20229700<br>C -5.50513500 -0.12359700 -1.67679000<br>C 0.20938500 -2.51033100 0.71230900<br>C -5.95228900 1.71165700 -0.16905300<br>C -0.31171400 -3.22642400 -0.51085900<br>C -6.33669300 0.88982300 -1.25543800<br>H -3.71807700 -2.05232100 -2.25714900<br>H -0.47225300 -0.88170600 1.97215400<br>H -4.46601600 2.17079800 1.29016800<br>H -1.71149100 -3.36927100 -2.07769000<br>H -5.79125000 -0.76576200 -2.50537800<br>H -0.00141100 -3.12480300 1.59907700<br>H -6.61132500 2.50841000 0.16217600<br>H 0.23128900 -4.11378200 -0.82752000<br>H -7.28733500 1.05959100 -1.75188500
T1	0 1<br>C -5.43414200 -1.42801600 0.00006700<br>C -5.58641100 -0.03351800 0.00007400<br>C -4.45905000 0.81282100 0.00004900<br>C -3.13369100 0.26938700 0.00001600<br>C -2.96468900 -1.19368800 0.00001000<br>C -4.14312000 -1.86907400 0.00003600<br>C -4.63749800 2.23973500 0.00005600<br>C -1.98680500 1.18028900 -0.00000800<br>C -2.22514700 2.59275000 0.00000000<br>C -3.57510800 3.08589100 0.00003300<br>C -1.14540200 3.49939300 -0.00002300<br>H -1.34367200 4.56910000 -0.00001700<br>C 0.17380600 3.03125800 -0.00005600<br>C 0.34255500 1.67029200 -0.00006300<br>C -0.60744700 0.69577900 -0.00004000<br>C -0.57392800 -0.74184300 -0.00004200<br>C -1.58242600 -1.47568600 -0.00002200<br>H -5.65152900 2.62819300 0.00008100<br>H -6.30094900 -2.08712600 0.00008600<br>H -6.58516600 0.39702700 0.00009800<br>H -3.72816900 4.16106400 0.00003900<br>H 1.00830200 3.72812900 -0.00007500<br>C 5.53293300 -1.81512700 -0.00010600<br>C 5.53408200 -0.41552900 -0.00001400<br>C 4.33939300 0.30901200 0.00004900

	C 3.12785800 -0.37071400 0.00001800<br>C 3.11133100 -1.77714900 -0.00007500<br>C 4.32284700 -2.49276100 -0.00013600<br>H 6.47492900 -2.34951000 -0.00015200<br>H 4.37166600 1.39154500 0.00012100<br>H 2.19490800 0.19723200 0.00007100<br>H 4.28843100 -3.57676400 -0.00020600<br>O 1.97120200 -2.50049300 -0.00010800<br>H 1.17790800 -1.92608600 -0.00007100<br>N 6.80481700 0.30118800 0.00001900<br>O 7.84665600 -0.36334400 -0.00005100<br>O 6.77579400 1.53710600 0.00008800<br>O -8.22242300 -3.22977700 0.00014800<br>H -8.54486800 -3.71504400 0.77018700<br>H -8.54498700 -3.71502200 -0.76985600
T2	0 1<br>C 2.66998100 3.33607400 -0.03693900<br>C 3.96686000 2.82194800 -0.07134300<br>C 4.19066300 1.42545400 -0.03551500<br>C 3.08331700 0.52252400 0.03663100<br>C 1.73074500 1.07878400 0.07719000<br>C 1.62752700 2.45140700 0.15773500<br>C 5.52156300 0.90523300 -0.11822900<br>C 3.33793000 -0.91341000 0.02868800<br>C 4.68653300 -1.38441400 -0.06009100<br>C 5.75970500 -0.43855100 -0.12976800<br>C 4.95692200 -2.77108900 -0.12165100<br>H 5.98926800 -3.11269100 -0.16038300<br>C 3.91433600 -3.70376900 -0.08965100<br>C 2.65506800 -3.20436000 0.13085600<br>C 2.24775700 -1.89567700 0.06413300<br>C 1.00687900 -1.21637000 0.08292300<br>C 0.80242300 0.00935300 0.08680700<br>H 6.34739000 1.60865000 -0.17296300<br>H 2.50421100 4.41030000 -0.06912800<br>H 4.82085000 3.49499500 -0.11159000<br>H 6.77627700 -0.81543800 -0.19429800<br>H 4.12500900 -4.76981900 -0.12208700<br>C -6.92118800 -0.91201800 -0.10753600<br>C -6.33464500 -2.17849100 0.07997000<br>C -4.96019500 -2.32147200 0.17832100<br>C -4.11083000 -1.20145900 0.09359200<br>C -4.71366100 0.06928600 -0.09561400<br>C -6.11065500 0.20484900 -0.19424300<br>H -7.99809900 -0.80957900 -0.18290400<br>H -6.96559800 -3.05968100 0.14934200<br>H -4.49683900 -3.29131400 0.32307700<br>H -6.52120100 1.19680900 -0.33711100

	O -2.79579400 -1.41648100 0.19621400<br>H -2.33241700 -0.55182800 0.12075700<br>N -3.91138700 1.26542700 -0.19057800<br>O -4.45755000 2.35119800 -0.35392100<br>O -2.66176700 1.14934100 -0.10086100<br>O -0.98313400 3.72136700 0.45984100<br>H -0.11523700 3.26502100 0.54689100<br>H -1.61015800 3.03019800 0.19666900
T3	0 1<br>C -1.35481300 -1.13890800 -1.08357000<br>C -1.47915900 -0.12681900 -0.12184500<br>C -2.75485100 0.30052200 0.32037600<br>C -3.94551900 -0.29039300 -0.21017500<br>C -3.82110800 -1.35476000 -1.21563400<br>C -2.52650700 -1.76103000 -1.41539500<br>C -2.86445900 1.36303500 1.27213900<br>C -5.24550100 0.21140300 0.20813900<br>C -5.30027300 1.27138000 1.16609000<br>C -4.08552000 1.82466000 1.67778400<br>C -6.55344200 1.80120500 1.55685100<br>H -6.57929600 2.60271100 2.29269600<br>C -7.73743600 1.30036900 1.01925000<br>C -7.69708300 0.19333800 0.18030600<br>C -6.50542200 -0.29571200 -0.33430900<br>C -6.18388100 -1.28427900 -1.29282400<br>C -5.09680500 -1.74842900 -1.68040500<br>H -1.95148100 1.79778700 1.66793000<br>H -0.37058800 -1.47718400 -1.39518900<br>H -0.57706800 0.29470800 0.31571700<br>H -4.15182100 2.63149900 2.40224600<br>H -8.68895500 1.72692200 1.33019100<br>O 2.12698600 -0.99436600 -0.89735700<br>O 1.92302200 0.67402000 0.48939100<br>N 2.60351000 -0.12368100 -0.16329900<br>C 4.77821400 -1.19384000 0.30401000<br>C 4.67093500 1.21454000 -0.32981500<br>C 6.21459700 -1.07846200 0.41777100<br>C 6.11184100 1.28176300 -0.23757200<br>C 4.06600400 -0.03120900 -0.05947300<br>C 6.83479500 0.14429300 0.13843000<br>C 4.17650600 -2.45532300 0.61285600<br>C 3.96545200 2.39257300 -0.73398200<br>C 6.97658200 -2.22141500 0.81733200<br>C 6.77301600 2.51474500 -0.53623200<br>C 4.94742500 -3.52315800 0.99879200<br>C 4.64291000 3.55197400 -1.01749600<br>C 6.36378500 -3.41315600 1.10029200<br>C 6.06177300 3.62208300 -0.91546500

	H 7.91717200 0.21291300 0.21591700<br>H 3.10375500 -2.57336300 0.52943400<br>H 2.88557000 2.37322200 -0.80615900<br>H 8.05534900 -2.11892600 0.89542400<br>H 7.85619800 2.54941500 -0.45970400<br>H 4.46830500 -4.47004100 1.22850900<br>H 4.08628100 4.43250200 -1.32376300<br>H 6.95028800 -4.27463100 1.40393200<br>H 6.57254200 4.55277100 -1.14188500<br>O -10.18766600 -1.07680000 -0.64527000<br>H -9.34818400 -0.67440200 -0.30118900<br>H -9.94372500 -1.96890000 -0.92247200
T4	0 1<br>C 2.07345100 3.14370800 -0.63178900<br>C 3.44386100 2.96086400 -0.45011000<br>C 3.97682900 1.66994400 -0.22044200<br>C 3.10557400 0.53741100 -0.17057000<br>C 1.67280900 0.75543000 -0.36825800<br>C 1.25083800 2.03078700 -0.70723700<br>C 5.37603100 1.49541300 0.01654100<br>C 3.66928100 -0.77489400 0.11213200<br>C 5.07505500 -0.89977600 0.34947000<br>C 5.90364600 0.26518300 0.29103500<br>C 5.63587800 -2.15579500 0.68449500<br>H 6.71136700 -2.24112100 0.82482500<br>C 4.82996300 -3.29564000 0.78518500<br>C 3.52661900 -3.13719600 0.39099200<br>C 2.82387600 -1.97145500 0.21013200<br>C 1.46498000 -1.62825200 0.01900400<br>C 1.02458700 -0.48948400 -0.21578800<br>H 6.01914800 2.36983700 -0.02361100<br>H 1.68295300 4.14366100 -0.80793800<br>H 4.12147500 3.81092000 -0.49785200<br>H 6.96855900 0.15253700 0.47186100<br>H 5.26157000 -4.26332600 1.02693400<br>C -5.71402500 -0.42793500 0.95421900<br>C -4.68402500 0.39039500 0.47352400<br>C -3.86404200 -0.01018200 -0.58520200<br>C -4.08525600 -1.25398300 -1.16761900<br>C -5.11260300 -2.08271200 -0.69530800<br>C -5.92892200 -1.66670200 0.36808000<br>H -6.32957900 -0.08441000 1.77621400<br>H -3.06565600 0.62748600 -0.95210300<br>H -3.45349600 -1.57817300 -1.99080500<br>H -6.71832600 -2.32322700 0.71696500<br>O -5.36922000 -3.30774600 -1.22980600<br>H -4.75126800 -3.49745300 -1.94901500<br>N -4.47054900 1.69136700 1.10248200<br>O -5.20032700 2.00736700 2.04570700

	O -3.56960400 2.41909300 0.66503900<br>O -1.53587100 2.09027800 -1.57607800<br>H -0.56689700 2.11513500 -1.37775300<br>H -1.97225100 2.52585400 -0.82889500
T5	0 1<br>C -0.00093800 -1.62982800 -0.01581800<br>C -0.11082400 -0.23282100 -0.02280500<br>C -1.38051500 0.39426500 -0.03669300<br>C -2.57900800 -0.38812300 -0.04021900<br>C -2.46874400 -1.85315900 -0.03874500<br>C -1.18058300 -2.30111500 -0.18592600<br>C -1.47614100 1.82121100 -0.00066000<br>C -3.87193500 0.27707800 0.01357700<br>C -3.91260400 1.70591900 0.04380900<br>C -2.69057800 2.44759000 0.03557200<br>C -5.15808400 2.37209800 0.13811500<br>H -5.17314200 3.46024800 0.15193300<br>C -6.34879400 1.65077200 0.20028400<br>C -6.32204500 0.27007800 0.04965000<br>C -5.13822300 -0.45194600 0.07580600<br>C -4.83122400 -1.83198400 0.09304500<br>C -3.74991200 -2.44488500 0.04440400<br>H -0.55765500 2.40050300 -0.00165000<br>H 0.97790000 -2.10033000 -0.02883000<br>H 0.79611200 0.36587900 -0.06504500<br>H -2.74584300 3.53221400 0.06232200<br>H -7.29447400 2.18334800 0.27754300<br>C 7.56849300 -1.37467900 0.15519500<br>C 8.22931400 -0.13543200 0.04575900<br>C 7.51716600 1.04709500 -0.07474600<br>C 6.11036300 1.03866200 -0.09070700<br>C 5.45437100 -0.21579500 0.02012300<br>C 6.18679300 -1.41181800 0.14199600<br>H 8.13599100 -2.29383300 0.24904300<br>H 9.31462300 -0.10164900 0.05554700<br>H 8.01541300 2.00660000 -0.15972600<br>H 5.64173600 -2.34402000 0.22358700<br>O 5.47990600 2.21245900 -0.20936600<br>H 4.51223700 2.02836000 -0.20328100<br>N 4.01594500 -0.29828900 0.01033900<br>O 3.47085300 -1.39645000 0.10534800<br>O 3.35643500 0.76707500 -0.09626800<br>O -8.83450500 -1.19056500 -0.19447500<br>H -8.60570000 -2.03586400 -0.60104800<br>H -7.98840400 -0.67269800 -0.18078300
T6	0 1<br>C -2.16766400 -3.48003400 -0.64113100<br>C -3.39352900 -2.82042600 -0.63132700

	C -3.45513300 -1.43365500 -0.41878400<br>C -2.24062700 -0.70092200 -0.22385000<br>C -0.97865700 -1.38406700 -0.25333200<br>C -0.97847800 -2.77661800 -0.45217200<br>C -4.71016800 -0.73077000 -0.38631600<br>C -2.30509900 0.70796600 0.00675200<br>C -3.56744000 1.37752900 0.03924300<br>C -4.76486000 0.61243200 -0.16714200<br>C -3.58797300 2.76160800 0.27466900<br>H -4.54139100 3.28188900 0.30295200<br>C -2.41013900 3.47825200 0.47409900<br>C -1.17494500 2.82715000 0.44151000<br>C -1.09892900 1.43825300 0.20739600<br>C 0.15226700 0.74659000 0.15262300<br>C 0.22992400 -0.60082800 -0.07514800<br>H -5.62239400 -1.30115400 -0.53863600<br>H -2.13306500 -4.55546800 -0.78774900<br>H -4.31568600 -3.37601600 -0.77875000<br>H -5.72106300 1.12854900 -0.14285000<br>H -2.44997600 4.54995600 0.65609300<br>C 4.13161900 -0.41556000 1.07271500<br>C 3.75606100 -0.01746500 -0.27540400<br>C 2.59802800 -0.38335300 -0.85255400<br>C 1.61364700 -1.26049500 -0.13863000<br>C 2.07070600 -1.76219500 1.25331300<br>C 3.33209500 -1.23073000 1.79483600<br>H 5.06793100 -0.03982500 1.46837100<br>H 2.35618300 -0.04158400 -1.85325800<br>H 1.52535400 -2.16602200 -0.75579200<br>H 3.59325200 -1.53916700 2.80194500<br>O 1.40103800 -2.58293900 1.86537300<br>H -0.04876200 -3.33178100 -0.42211500<br>N 4.69307300 0.83945900 -1.02696600<br>O 5.73106700 1.17501800 -0.45200000<br>O 4.39126200 1.16878200 -2.17377500<br>O 0.00673300 3.49324900 0.63111900<br>H -0.16159100 4.43408300 0.77093000<br>H 1.05465400 1.33147300 0.29368600
T7	0 1<br>C -2.16766400 -3.48003400 -0.64113100<br>C -3.39352900 -2.82042600 -0.63132700<br>C -3.45513300 -1.43365500 -0.41878400<br>C -2.24062700 -0.70092200 -0.22385000<br>C -0.97865700 -1.38406700 -0.25333200<br>C -0.97847800 -2.77661800 -0.45217200<br>C -4.71016800 -0.73077000 -0.38631600<br>C -2.30509900 0.70796600 0.00675200<br>C -3.56744000 1.37752900 0.03924300

	C -4.76486000 0.61243200 -0.16714200<br>C -3.58797300 2.76160800 0.27466900<br>H -4.54139100 3.28188900 0.30295200<br>C -2.41013900 3.47825200 0.47409900<br>C -1.17494500 2.82715000 0.44151000<br>C -1.09892900 1.43825300 0.20739600<br>C 0.15226700 0.74659000 0.15262300<br>C 0.22992400 -0.60082800 -0.07514800<br>H -5.62239400 -1.30115400 -0.53863600<br>H -2.13306500 -4.55546800 -0.78774900<br>H -4.31568600 -3.37601600 -0.77875000<br>H -5.72106300 1.12854900 -0.14285000<br>H -2.44997600 4.54995600 0.65609300<br>C 4.13161900 -0.41556000 1.07271500<br>C 3.75606100 -0.01746500 -0.27540400<br>C 2.59802800 -0.38335300 -0.85255400<br>C 1.61364700 -1.26049500 -0.13863000<br>C 2.07070600 -1.76219500 1.25331300<br>C 3.33209500 -1.23073000 1.79483600<br>H 5.06793100 -0.03982500 1.46837100<br>H 2.35618300 -0.04158400 -1.85325800<br>H 1.52535400 -2.16602200 -0.75579200<br>H 3.59325200 -1.53916700 2.80194500<br>O 1.40103800 -2.58293900 1.86537300<br>H -0.04876200 -3.33178100 -0.42211500<br>N 4.69307300 0.83945900 -1.02696600<br>O 5.73106700 1.17501800 -0.45200000<br>O 4.39126200 1.16878200 -2.17377500<br>O 0.00673300 3.49324900 0.63111900<br>H -0.16159100 4.43408300 0.77093000<br>H 1.05465400 1.33147300 0.29368600
T8	0 1<br>C -2.88494600 3.13092600 1.39806600<br>C -4.17972500 2.80675100 1.17658900<br>C -4.56661400 1.54429100 0.56622500<br>C -3.58103600 0.57150900 0.31967900<br>C -2.17443800 0.83299600 0.60580400<br>C -1.78640800 2.19216500 1.10126200<br>C -5.90608200 1.31737600 0.14805400<br>C -3.92298700 -0.59131700 -0.43387200<br>C -5.26773400 -0.80417600 -0.85616100<br>C -6.25410800 0.17051400 -0.52894600<br>C -5.57690700 -1.97297800 -1.59768500<br>H -6.60155000 -2.12900500 -1.92507400<br>C -4.59819400 -2.90199100 -1.91641600<br>C -3.26785500 -2.69191000 -1.51431800<br>C -2.92412100 -1.55548800 -0.78017900<br>C -1.63612300 -1.35720600 -0.14594600

	C -1.23092000 -0.12042000 0.31603400<br>H -6.65768900 2.07193000 0.36331000<br>H -2.59702200 4.09505100 1.80528700<br>H -4.96867800 3.51945200 1.40702200<br>H -7.28342700 0.00303900 -0.83332200<br>H -4.85429200 -3.78690100 -2.49025200<br>O 6.22769400 -1.95212900 -0.86140200<br>O -0.61407300 2.51152900 1.31857300<br>N 5.80505100 -0.80427000 -1.05280700<br>C 3.83224900 -1.17721000 0.50852200<br>C 4.25597300 0.94678000 -0.72821100<br>C 2.65543000 -0.56420800 1.07685400<br>C 3.06374600 1.51504600 -0.15225500<br>C 4.62573300 -0.39805300 -0.39694600<br>C 2.29663600 0.74913800 0.72981300<br>C 4.11959600 -2.52309000 0.89399800<br>C 5.01677800 1.76509400 -1.61815800<br>C 1.84087200 -1.29543900 1.99496000<br>C 2.67210400 2.85136500 -0.48377800<br>C 3.30197300 -3.20047800 1.77531400<br>C 4.61295700 3.04730900 -1.90984000<br>C 2.15253600 -2.58989200 2.33948700<br>C 3.43044600 3.60252900 -1.34331300<br>H 1.38331800 1.17529600 1.13829900<br>H 4.99189900 -3.00730500 0.48397600<br>H 5.91651400 1.35625300 -2.05826700<br>H 0.97839500 -0.79171000 2.42270700<br>H 1.76316100 3.24323700 -0.03668800<br>H 3.55053600 -4.22262200 2.04678500<br>H 5.20958600 3.64999900 -2.58914100<br>H 1.53552400 -3.13829900 3.04560600<br>H 3.13633400 4.61700500 -1.59525700<br>O -0.90035800 -2.46241300 0.06795900<br>H -0.06083500 -2.19127600 0.48874200<br>H -2.49620600 -3.40975300 -1.77294500
T9	0 1<br>C 1.73199500 3.05861100 -1.41783900<br>C 2.61188000 2.24876400 -2.12961800<br>C 3.05179200 1.01949600 -1.60825000<br>C 2.58109500 0.59015500 -0.32198700<br>C 1.66943300 1.43177000 0.38126400<br>C 1.26245000 2.65744700 -0.16738400<br>C 3.96394500 0.18919300 -2.33706200<br>C 3.03567600 -0.65734100 0.21977200<br>C 3.95597500 -1.45846900 -0.53496500<br>C 4.39421800 -0.99327800 -1.82463200<br>C 4.43557500 -2.67708000 -0.03231400<br>H 5.12819700 -3.28440000 -0.60685600

	C 4.00346100 -3.10177900 1.23234600<br>C 3.12887600 -2.25596200 1.82558600<br>C 2.52513700 -1.07026300 1.58144200<br>C 1.61566300 -0.20460500 2.21094100<br>C 1.20575200 0.95282400 1.65710500<br>H 4.30722900 0.52553000 -3.31117300<br>H 1.40859400 4.00719000 -1.83531000<br>H 2.97593100 2.56210700 -3.10405800<br>H 5.08687100 -1.61670100 -2.38181500<br>H 4.33673400 -4.03017900 1.68507000<br>C -5.03195700 0.58209200 -0.29482900<br>C -4.51939200 -0.71956700 -0.33713100<br>C -3.24386700 -1.01234500 0.15156500<br>C -2.46817000 0.00804700 0.68914600<br>C -2.97055900 1.32159600 0.73768600<br>C -4.25728100 1.59958200 0.24249600<br>H -6.02473900 0.77696500 -0.68128800<br>H -2.87478600 -2.02952600 0.10551800<br>H -1.47539200 -0.21043700 1.07060600<br>H -4.62655500 2.61825300 0.28984000<br>O -2.26723400 2.35677900 1.24690700<br>H -1.38576100 2.07763500 1.56961100<br>N -5.33166500 -1.79185300 -0.90339100<br>O -6.45632700 -1.50601100 -1.32782200<br>O -4.85354700 -2.93125100 -0.93000200<br>O 0.28064300 1.75860500 2.31059500<br>H 0.16550400 1.37790200 3.19699800<br>H 0.58135700 3.29032000 0.38916000
T10	0 1<br>C -3.00215200 3.19067500 0.40694000<br>C -4.05229300 2.27984300 0.31217000<br>C -3.80552200 0.90895200 0.13646500<br>C -2.45056000 0.46252600 0.05774100<br>C -1.37238300 1.39053900 0.15366700<br>C -1.67673800 2.75617300 0.32891200<br>C -4.86116700 -0.06080400 0.03656100<br>C -2.16537100 -0.92681000 -0.11446900<br>C -3.23782600 -1.86814800 -0.21055800<br>C -4.59126600 -1.38643700 -0.12933600<br>C -2.93736400 -3.23064300 -0.38023000<br>H -3.75202300 -3.94645500 -0.45135500<br>C -1.61523500 -3.66655300 -0.45537000<br>C -0.55858400 -2.75971700 -0.36482100<br>C -0.81562800 -1.38958500 -0.19449400<br>C 0.23435300 -0.40698800 -0.09347000<br>C -0.01971000 0.92012600 0.08174500<br>H -5.88930600 0.28667700 0.09769600<br>H -3.21267700 4.24948300 0.54232300

	H -5.07725000 2.63445200 0.37566100<br>H -5.40075400 -2.10772500 -0.20222000<br>H -1.40310300 -4.72387900 -0.58412400<br>C 4.89114400 1.10542900 -1.50792500<br>C 3.72963500 1.06442700 -2.28913000<br>C 2.58511500 0.41967400 -1.82547600<br>C 2.57391200 -0.17412400 -0.55769300<br>C 3.73823800 -0.11365500 0.22239300<br>C 4.89885300 0.50105800 -0.25505900<br>H 5.78411100 1.59990400 -1.87532300<br>H 3.72020400 1.52185400 -3.27376800<br>H 1.69269300 0.35599300 -2.43848200<br>H 5.78328600 0.50126800 0.37125000<br>O 1.51655800 -0.93231500 -0.13662700<br>H 0.46729600 -3.10470000 -0.41820200<br>N 3.77694300 -0.68421300 1.57713200<br>O 4.83549600 -1.20895700 1.93304300<br>O 2.77472900 -0.57460500 2.27931600<br>O -0.61864500 3.62472100 0.41382000<br>H 0.78814000 1.63407300 0.18122600<br>H -0.94100000 4.52205400 0.56761700
T11	0 1<br>C -1.65770400 0.38918500 0.92251900<br>C -2.64519300 0.95072400 1.72521100<br>C -4.01155700 0.79421000 1.43801700<br>C -4.39301500 0.03236500 0.28283400<br>C -3.37509400 -0.53726000 -0.52909000<br>C -2.01439200 -0.35727000 -0.20662100<br>C -5.02624000 1.37250700 2.26545800<br>C -5.78489900 -0.14372900 -0.03542600<br>C -6.77154400 0.45354500 0.82045600<br>C -6.34268500 1.20922000 1.96941300<br>C -8.13916100 0.30988800 0.55048400<br>H -8.86986200 0.76978400 1.21099100<br>C -8.55113900 -0.42284400 -0.56526600<br>C -7.58822900 -0.98242400 -1.36437400<br>C -6.22566500 -0.93038300 -1.23160600<br>C -5.09960100 -1.41719700 -1.91435400<br>C -3.79236600 -1.29839300 -1.68333300<br>H -4.72270100 1.94614400 3.13720400<br>H -0.60845600 0.52434500 1.16763800<br>H -2.35756100 1.52719000 2.60038600<br>H -7.10922700 1.64991600 2.60015600<br>H -9.60891100 -0.54265800 -0.78796100<br>O 1.57597100 -0.16988400 -0.20001500<br>O 2.50918700 -1.72376100 -1.40672600<br>N 2.57570100 -0.74423200 -0.66889800<br>C 4.13112500 1.16460500 -0.49310300

	C 4.85052600 -1.12350000 0.20006500<br>C 5.44375600 1.65885100 -0.14386300<br>C 6.13927700 -0.58242400 0.56835500<br>C 3.89362000 -0.21642300 -0.30919500<br>C 6.39758900 0.77959800 0.38007600<br>C 3.19125500 2.09028000 -1.04799200<br>C 4.61861100 -2.51773900 0.42486000<br>C 5.74693600 3.04239700 -0.34395300<br>C 7.13069600 -1.45151000 1.12322800<br>C 3.52906500 3.40838200 -1.22997400<br>C 5.59777300 -3.31254500 0.96727900<br>C 4.81682100 3.89826700 -0.87131300<br>C 6.87152900 -2.78211300 1.31814400<br>H 7.37618300 1.16908700 0.65019200<br>H 2.19928700 1.75310800 -1.31828500<br>H 3.66726200 -2.95622000 0.15341500<br>H 6.73752300 3.39631900 -0.07235000<br>H 8.09435200 -1.02749900 1.39127100<br>H 2.79822200 4.08982500 -1.65465300<br>H 5.39639700 -4.36718900 1.12867200<br>H 5.05581100 4.94636500 -1.02164500<br>H 7.62919000 -3.43441100 1.74086100<br>O -1.10028000 -0.93284300 -1.03137200<br>H -0.18953500 -0.74475400 -0.73812600<br>H -3.03746300 -1.74744000 -2.32219900

PYTHON script of adsorption energy calculation for Dimers, Trimers and complexes (Ex: 4Nitrophenol)

```
# Import libraries

import pandas as pd

# Import data from excel file

df = pd.read_excel ('ads.xlsx','4np')

# Indexation

df.set_index(['configuration'], inplace=True)

# Specify the column

df1 = df ['t']
```

```
df2 = df ['b']

df3 = df ['p']

# Function for calculating energy adsorption

def global_parameters (df1, df2, df3):

# Mathematical equations of calculation

    df ['E'] = (df.t - ( df.b + df.p ))

    return df

print (global_parameters (df1, df2, df3))
```

Where 'E' refers to adsorption energy, 't' the total energy of optimized structure after the adsorption and 'b' is the energy of the bulk corresponding to each specific onfiguratand 'p' is the energy of the pollutants.
